# Protein Engineering of an Artificial P450BM3 Peroxygenase System Enables Highly Selective *O*-Demethylation of Lignin Monomers

**DOI:** 10.3390/molecules27103120

**Published:** 2022-05-13

**Authors:** Maosheng Li, Hengmin Miao, Yanqing Li, Fang Wang, Jiakun Xu

**Affiliations:** 1 School of Food Science and Engineering, Qilu University of Technology (Shandong Academy of Sciences), Jinan 250353, China; 17806022501@163.com; 2 Key Lab of Sustainable Development of Polar Fisheries, Ministry of Agriculture and Rural Affairs, Yellow Sea Fisheries Research Institute, Chinese Academy of Fishery Sciences, Lab for Marine Drugs and Byproducts of Pilot National Lab for Marine Science and Technology, Qingdao 266071, China; m19862524817@163.com (H.M.); liyanqing527@163.com (Y.L.); wendywf2002@163.com (F.W.)

**Keywords:** protein engineering, peroxygenase, lignin monomers, dual-functional small molecule, *O*-demethylation

## Abstract

The *O*-demethylation of lignin monomers, which has drawn substantial attention recently, is critical for the formation of phenols from aromatic ethers. The P450BM3 peroxygenase system was recently found to enable the *O*-demethylation of different aromatic ethers with the assistance of dual-functional small molecules (DFSM), but these prepared mutants only have either moderate *O*-demethylation activity or moderate selectivity, which hinders their further application. In this study, we improve the system by introducing different amino acids into the active site of P450BM3, and these amino acids with different side chains impacted the catalytic ability of enzymes due to their differences in size, polarity, and hydrophobicity. Among the prepared mutants, the combination of V78A/F87A/T268I/A264G and Im-C6-Phe efficiently catalyzed the *O*-demethylation of guaiacol (TON = 839) with 100% selectivity. Compared with NADPH-dependent systems, we offer an economical and practical bioconversion avenue.

## 1. Introduction

**Figure 1:**
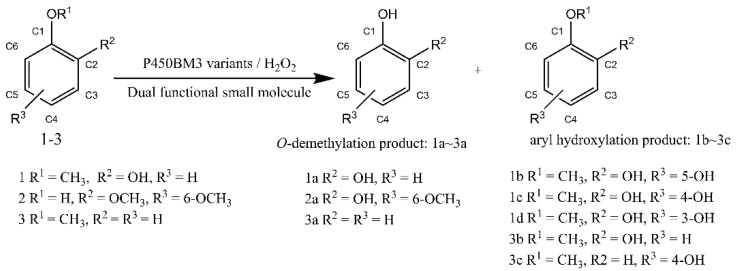
Oxidation of aromatic ethers catalyzed by P450BM3 mutants in the presence of N-(ω-imidazol-1-yl hexanoyl)-L-phenylalanine (Im-C6-Phe) or N-(ω-imidazol-1-yl valeryl)-L-phenylalanine (Im-C5-Phe).

**Figure 2:**
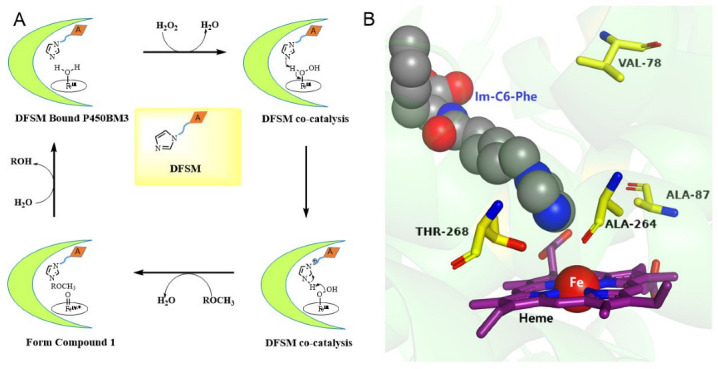
(**A**) Proposed catalytic cycle of the artificial P450BM3 peroxygenase system assisted by a DFSM. (**B**) The active site of P450BM3 containing N-(ω-imidazol-1-yl hexanoyl)-L-phenylalanine (Im-C6-Phe. PDB code: 7EGN [22]); the residues located at proximal positions of heme are highlighted using pymol [23].

As the only renewable aromatic carbon feedstock, lignin has significant and largely unrealized potential as a source for the sustainable production of fuel and bulk high-value chemicals [1]. Because of its heterogeneous and recalcitrant constitution, the catabolic process of lignin is hampered by a lack of practical biological lignin-catabolic pathways [2]. In the catabolism of lignin-derived compounds, the *O*-demethylation of aromatic ethers is a crucial step in the formation of phenols, which are precursors to manufacturing diols by aromatic ring-opening reactions [3,4,5,6]. Recent studies on the enzymatic demethylation of lignin-derived compounds and their model compounds have primarily focused on white-rot, soft-rot, and brown-rot fungi and some bacteria [7,8]. Although some valuable enzymes have been identified for the demethylation of lignin and its derivatives, there is still a large demand for developing new *O*-demethylases for the demethylation of lignin and its derivatives either from natural sources or laboratory evolution [9,10,11,12,13,14,15,16,17]. Previously, an artificial P450BM3 peroxygenase system, which has a dual-functional small molecule (DFSM) installed in the active pocket, was developed to perform an H_2_O_2_-dependent monooxygenation of non-native substrates [18,19,20]. Through the dual-functional small molecule (DFSM)-facilitated P450BM3 peroxygenase system, we have recently achieved regioselective *O*-demethylation of various aromatic ethers with H_2_O_2_ as an oxidant, opening up alternative avenues for lignin conversion (Figure 1 and Figure 2A) [21]. The above H_2_O_2_-dependent P450 enzyme can catalyze the demethylation of guaiacol and various lignin-related monomers, and both arrangements of amino acid residues in the active site and DFSM structure could affect the substrate orientation at the active site. Although the DFSM-facilitated P450BM3 peroxygenase system exhibited catalytic ability, the catalytic system’s selectivity remained unsatisfactory and required further enhancement. For example, F87A and F87G of P450BM3 could catalyze the reaction (F87A, TON: 883 and F87G, TON: 731), but the selectivities of F87A and F87G were only 61% and 68%, respectively [21]. From the perspective of economic production, this system should be more economical and efficient for industrial application.

To further enhance the demethylation catalytic ability of the P450BM3-DFSM peroxygenase system, the active site was rationally designed, and different amino acids were introduced to positions such as Ala 264, Thr 268 in the heme center and Val 78 on the substrate channel (Figure 2B), which are expected to provide more options for controlling the regioselectivity through protein engineering. As shown in Figure 2B, Ala 264 and Thr 268 were very located close to both heme and the imidazolyl group of Im-C6-Phe. Residue Ala 264 and residue Thr 268 were mutated to several amino acids to check the influence of size, polarity, and hydrophobicity on the catalytic ability. Residue Val 78 located in the substrate channel was replaced with smaller residues of glycine and alanine to provide space to accommodate the substrate and H_2_O_2_. Herein, we developed several P450BM3 mutants, some of which can efficiently realize the efficient conversion of lignin in the presence of DFSM/H_2_O_2_. Compared with the native NADPH-dependent P450BM3, the DFSM-facilitated P450BM3 peroxygenase system is much more economical and practical due to the use of inexpensive H_2_O_2_ as an oxidant as well as the absence of other coenzymes, which serves as an alternative practical method for lignin bioconversion [20]. The enzymatic activities of different mutants were screened in the presence of DFSM with guaiacol (1) as the model substrate. We then calculated the turnover number (TON) of different mutants of P450BM3 through the amount of formed products as checked by HPLC.

## 2. Results and Discussions

### 2.1. O-demethylation of Guaiacol Catalyzed by the P450BM3 Peroxygenase System

First, we performed the reactions involving guaiacol in the presence of DFSM using P450BM3 mutants (Table S1 and Figure S1, Supplementary Materials). The results are summarized in Table 1. Single mutants F87A and F87G could catalyze the demethylation of guaiacol (1) to catechol (1a) as the primary product with a turnover number of 539 and 495 in our previous study, and the selectivities were 61% and 68%, respectively (Table 1) [21]. Then, we examined the effects of mutations at position Thr 268 for the activity and selectivity of P450BM3 by mutation of threonine 268 to proline and leucine. In the presence of Im-C6-Phe, F87A/T268P could catalyze guaiacol oxidation with catechol (1a) as a major product (TON: 321), and the selectivity of *O*-demethylation was increased to 86%. The selectivity could be further enhanced to 100% by combining F87A/T268L and Im-C6-Phe; however, F87A/T268L exhibited low catalytic activity (TON: 10) and was considerably lower than F87A/T268A (TON: 96), which showed the highest activity in mutants of 100% *O*-demethylation mentioned in the previous study (Table 1) [21].

We assumed that residues with a smaller side chain at position 78 affect the binding of the foreign substrate in the active site of P450BM3; thus, we devised several double mutants (F87A/V78A and F87G/V78A) by further mutation of the amino acid at position 78 from valine to alanine based on F87A and F87G, respectively. F87A/V78A and F87G/V78A exhibited better regioselectivity (92% and 86%, respectively) for guaiacol than F87A and F87G (61% and 68%, respectively), but the demethylation activity had slightly reduced the TON of 448 and 285, respectively.

Because of the improved regioselectivity and TON of double mutants F87A/V78A and F87A/T268P for guaiacol, we further examined the effect of mutation at position Thr 268 on the activity of P450BM3 by introducing isoleucine and proline to prepare the triple mutants using F87A/V78A and F87A/V78G as templates (Table 1). The total TONs of F87A/V78A/T268I and F87A/V78G/T268I for guaiacol oxidation were significantly increased to 1272 and 738, respectively; however, the *O*-demethylation selectivity decreased to 34% and 25%, respectively. F87A/V78A/T268P and F87A/V78G/T268P gave the total TON of 280 and 441 and *O*-demethylation selectivity of 62% and 80%, respectively (Table 1). These results indicated that the mutation of threonine to isoleucine at position Thr 268 could greatly enhance the catalytic activity of P450BM3.

Owing to the high catalytic activity of F87A/V78A/T268I, we selected it as the parent template to perform the next round of mutations. To further improve *O*-demethylation selectivity, we introduced several amino residues (glycine, serine, cystine, and isoleucine) to assess the influence of size, polarity, and hydrophobicity of amino acids at position Ala 264 on the catalytic ability. When alanine was replaced with smaller glycine, the combination of F87A/V78A/T268I/A264G and Im-C6-Phe showed the best catalytic TON of 839 to guaiacol with 100% selectivity (Table 1). In contrast, all other involved mutations at position 264 (F87A/V78A/T268I/A264S, F87A/V78A/T268I/A264C, F87A/V78A/T268I/A264I) exhibited no catalytic activity for guaiacol oxidation in the presence of Im-C6-Phe (Table S1). We speculated that amino acids with a larger side chain at 264 will hinder the access of guaiacol, which could explain the abrupt disappearance of activity. This speculation was also supported by F87A/V78A/T268I/A264G, which has a smaller side chain than native alanine at position 264.

Because of these mutants with favorable properties, we performed some mutations using them as templates. As expected, all prepared mutants exhibited great *O*-demethylation activity. Among these mutants, F87A/T268P/A264G and F87A/V78A/A264G/T268A exhibited 100% selectivity to guaiacol demethylation in the presence of Im-C6-Phe; however, these mutants did not exhibit higher TON numbers (269 and 154, respectively) (Table 1).

We also performed reactions of P450BM3 in the presence of N-(ω-imidazol-1-yl pentanoyl)-L-phenylalanine (Im-C5-Phe), but no oxidized products were observed in the above mutants, indicating that the structure of DFSM is essential to the catalytic activity and regioselectivity of the P450BM3 peroxygenase system (Table S1).

### 2.2. O-demethylation of Syringol Catalyzed by the P450BM3 Peroxidase System

Next, we tried to expand the substrate scope of the aromatic *O*-demethylation catalyzed by the DFSM-facilitated peroxygenase system to exploit its potential for syringol (S-lignin) bioconversion (Table S2 and Figure S2). The results are summarized in Table 2. These reactions for syringol only gave the mono-demethylated product (2a), and no aromatic hydroxylation product was identified. Among all mutants discussed above, F87A/T268P exhibited the highest *O*-demethylation activity for syringol (2a, TON: 238), which was 44% higher than F87G/T268V (2a, TON: 165) (Table 2) reported in our previous study [21].

F87A/V78A/T268I/A264G with the best catalytic ability to guaiacol demethylation did not show satisfactory catalytic ability for syringol in the presence of Im-C6-Phe (2a, TON: 73) (Table 2). Except for F87A/T268P, there are several mutants that show good catalytic activity to syringol. F87A/V78A/T268I and F87A/V78G/T268I, respectively, gave the TON of 200 and 208 to the *O*-demethylation of syringol, which were also higher than those of the previous study. We also examined the combinations of P450BM3 mutants and Im-C5-Phe for syringol demethylation. These reactions gave no oxidized products of syringol, and similar trends were also found in the oxidation of guaiacol using the combination of P450BM3 mutants and Im-C5-Phe, which suggests that the length of Im-C5-Phe was insufficient for the oxidation of lignin monomers (Table S2).

### 2.3. O-demethylation of Anisole Catalyzed by the P450BM3 Peroxidase System

Furthermore, the reactions of anisole (3) were performed in the presence of DFSM using P450BM3 mutants (Table S3 and Figure S3). The results are summarized in Table 3. Unlike substrates 1–2, the mutants prepared in this study did not exhibit improved TON to anisole compared with that in our previous study, including F87A/V78A/T268I/A264G and F87A/T268P that exhibited excellent activity to guaiacol and syringol, respectively. Among these mutants, F87A/V78A/T268I gave good catalytic activity against anisole demethylation (3a: 265) in the presence of Im-C6-Phe, similar to the activity of F87A/T268I (3a: 262) reported in our previous study (F87A/T268I exhibited the best demethylation activity in the presence of Im-C6-Phe) [21]. Notably, the mutation of valine to alanine at position 78 of F87A/T268I P450BM3 did not exhibit a negative effect on the demethylation of anisole, whereas the mutation liberated its activity to guaiacol demethylation. This discovery demonstrated that one typical mutant could catalyze several reactions of substrates possessing similar structures. In addition, F87A/V78A/T268I/A264S showed the catalytic TON of 132 to anisole, which was different from its demethylated reaction to guaiacol (no catalytic product was identified). This difference may be due to the smaller size of anisole that only has one substitute group in the benzene ring compared with guaicol, which has two substitute groups. The result corresponded to previous speculations about mutations to substrate docking at position 264 in 2.1. The combinations of P450BM3 mutants and Im-C5-Phe for anisole demethylation were also performed. As with substrates 1–2, these reactions gave no oxidized products of anisole.

### 2.4. MS Assay of Reaction Products

**Figure 3:**
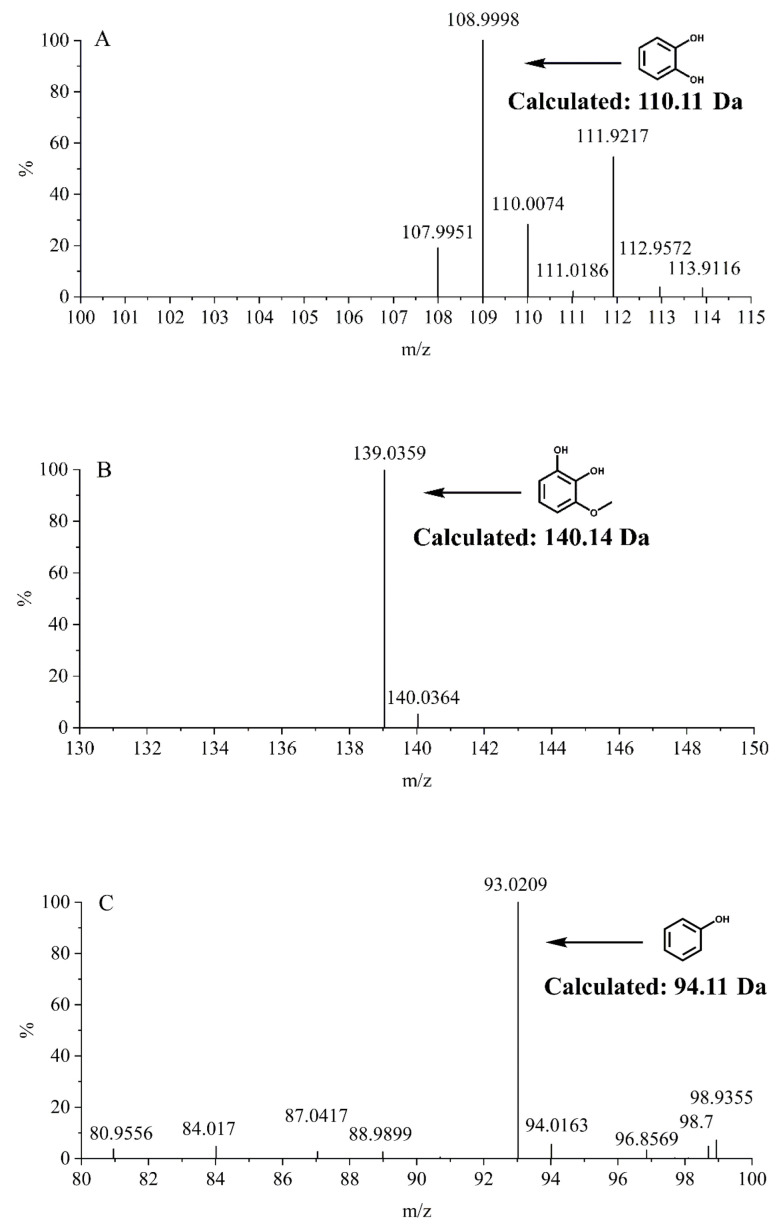
MS spectra of enzymatic reaction products of guaiacol, syringol and anisole by F87A/V78A/T268I/A264G P450BM3. (**A**) catechol (109 *m/z*); (**B**) 3-methoxycatechol (139 *m/z*); (**C**) phenol (93 *m/z*).

To further identify the *O*-demethylated products of guaiacol, syringol and anisole catalyzed by F87A/V78A/T268I/A264G P450BM3, we performed UPLC-MS studies for the solution after reaction. As shown in Figure 3, the mass of *m/z* 109.00 (Figure 3A), 139.03 (Figure 3B), and 93.02 (Figure 3C) for new peaks were generated after the reaction, which corresponded to the mass of catechol (Figure 3A), 3-methoxycatechol (Figure 3B), and phenol (Figure 3C), respectively. Based on these observations, we confirmed that demethylation had taken place in these reactions.

### 2.5. ^1^ H NMR Assay of Reaction Products

The *O*-demethylation products of different aromatic ethers were confirmed by ^1^H NMR. These spectrums were shown in Supplementary Materials (guaiacol: Figure S6; syringol: Figure S7; anisole: Figure S8).

## 3. Materials and Methods

### 3.1. Materials

A Q5 site-directed mutagenesis kit was purchased from New England Biolabs. Guaiacol, 3-methoxycatechol, catechol, anisole, 4-methoxyphenol, phenol, 2,6-dimethoxyphenol, pyrogallol, and 3-methoxycatechol were purchased from TCI. 2-methoxyhydroquinone and 4-methoxybenzene-1,3-diol were purchased from Bidepharm. All chemicals and solvents were used as purchased without further purification. Dual-functional small molecules including N-(ω-imidazol-1-yl hexanoyl)-L-phenylalanine (Im-C6-Phe) and N-(ω-imidazol-1-yl pentanoyl)-L-phenylalanine (Im-C5-Phe) were kind gifts from Dr. Zhiqi Cong at Qingdao Institute of Bioenergy and Bioprocess Technology, Chinese Academy of Sciences.

### 3.2. Methods

#### 3.2.1. Expression and Purification of P450BM3

The P450 BM3 heme domain (BMP, residues 1–455) and a his-tag at the N-terminus was ligated with the vector pET-28a (+) digested with the same restriction enzymes. The pET-28a (+) vectors containing BMP and its variants were transformed into *Escherichia coli* BL21(DE3) cells, and the cells were cultivated in LB medium containing 50 µg/mL kanamycin. The cultures were grown at 37 °C with vigorous shaking (≈200 rpm). When the OD600 of the cultures reached 0.8~1.0, the temperature was cooled to 30 °C, and the expression was induced by the addition of IPTG (1 mM) and δ-aminolevulinic acid hydrochloride (0.5 mM). Following 16–20 h of expression, the cells were harvested by centrifugation and stored at −20 °C. Cell pellets were resuspended in ice-cold buffer A (100 mM KPi, 100 mM NaCl, imidazole (20 mM), pH 7.4) and lysed by sonication. Cell debris was removed by centrifugation for 30 min at 20,000 *g*. Purification was completed by Ni-NTA metal-affinity chromatography. The crude cell extraction was applied to a 5 mL bed volume column pre-equilibrated with buffer A. Nonspecifically bound proteins were washed from the column with 5 column volumes of buffer A containing 30 mM imidazole. The bound protein was eluted with buffer B (100 mM KPi, 100 mM NaCl, imidazole (200 mM), pH 7.4). The purified protein solution was exchanged with buffer C (100 mM KPi, 100 mM NaCl, pH 7.4). BMP and its variants fractions were checked for purity by SDS-PAGE (Figure S4), concentrated by ultrafiltration and frozen in buffer C plus 50% glycerol at −20 °C.

The formation of a ferrous CO complex was confirmed by UV-visible spectral change through the reduction in the ferric heme of the P450BM3 mutants by the addition of Na_2_S_2_O_4_ in the presence of carbon monoxide (CO) (Figure S5) [24]. The concentrations of P450BM3 and its variants were measured by hemochrome binding assay [25]. A pyridine solution was made by combining pyridine (1.75 mL) and 1 M aqueous of NaOH (0.75 mL). The solution was mixed at room temperature and then centrifuged for 30 s at 5000 rpm to remove excess aqueous base. To a cuvette containing 0.75 mL of protein solution in phosphate buffer (0.1 M, pH 8.0), 0.25 mL of the pyridine solution was added followed by 2 mg of sodium dithionite. A UV-vis spectrum was recorded immediately. Hemoprotein concentration was determined from the absorbance of the hemochrome complex using extinction coefficients of ε418 = 196 mM^−1^ cm^−1^. Absorbance was assigned as the difference between the peak max at 418 nm and the baseline at 420 nm as determined by extrapolating from two points on either side of the hemochrome peak (390 nm and 450 nm).

#### 3.2.2. Mutagenesis

PCR reaction system: 12.5 μL of Q5 Hot Start High-Fidelity 2X Master Mix, 1.25 μL forward primer (0.5 μM), 1.25 μL reverse primer (0.5 μM), template DNA (1–25 ng) and nuclease-free water were subsequently transferred into a 100 μL of EP tube, and PCR amplification program was set by Q5 site-directed mutagenesis kit protocol. After ligation and transformation, the mutants prepared were verified by DNA sequencing. The primers used are shown in Table S4.

#### 3.2.3. General Procedure for *O*-demethylation of Aromatic Ethers

P450BM3 (0.5 μM) was transferred to a glass sample bottle containing 0.1 M, pH 8.0 phosphate buffer, aromatic ether compounds (4 mM, dissolved in methanol) and dual functional small molecule (DFSM) (500 μM, dissolved in pH 8.0 phosphate buffer). The reaction was initiated by the addition of H_2_O_2_ (30 mM, dissolved in pH 8.0 phosphate buffer). The reaction mixture was incubated in a water bath at 25 °C for 30 min. The reaction was stopped by the addition of dilute HCl aqueous (1 M) and neutralized with an equal volume of KCl (1 M). The products were directly analyzed by HPLC (see below).

#### 3.2.4. Product Analysis by HPLC

All measurements were performed two or more times in the presence of DFSM. A minimum of 6 calibration levels was used with an r^2^ coefficient of 0.9992 or better for each analyte.

Analysis of samples was performed on an Agilent Technologies 1200 Series equipped with PDA. Each sample and standard were injected at a volume of 20 μL onto a Waters sunfire^TM^ C18 5.0 μm (4.6 mm × 150 mm column). The column temperature was maintained at 30 °C, and the buffers used to separate the analytes of interest were 0.05% acetic acid in water (A)/acetonitrile (B). The specific elution programs for the reactions of different substrates were as follows:

Guaiacol: The system was run with A–B (83:17) and under these conditions 2-methoxyhydroquinone eluted at 5.587 min, 4-methoxybenzene-1,3-diol at 6.966 min, catechol at 9.539 min, 3-methoxycatechol at 10.402 min and the substrate at 25.174 min. The flow rate was held constant at 0.6 mL min-1, resulting in a run time of 30 min. The calibration curve concentration for each reaction product and substrate varied within the ranges of 5–400 µM and 0.05–4 mM, respectively. The detection wavelength for analysis of the analytes of interest was 210 nm.

2,6-dimethoxyphenol: The system was run with A–B (80:20), and under these conditions, pyrogallol eluted at 5.345 min, 3-methoxycatechol at 10.346 min and the substrate at 22.734 min. The flow rate was held constant at 0.5 mL min^−1^, resulting in a run time of 30 min. The calibration curve concentration for each reaction product and substrate varied within the ranges of 5–400 µM and 0.05–4 mM, respectively. The detection wavelength for analysis of the analytes of interest was 210 nm.

Anisole: The system was run with A–B (70:30), and under these conditions, 4-methoxyphenol eluted at 8.144 min, phenol at 9.632 min, guaiacol at 10.513 min and the substrate at 39.327 min. The flow rate was held constant at 0.6 mL min^−1^, resulting in a run time of 45 min. The calibration curve concentration for each reaction product and substrate varied within the ranges of 5–400 µM and 0.05–4 mM, respectively. The detection wavelength for analysis of the analytes of interest was 210 nm.

#### 3.2.5. UPLC-MS Assay of Reaction Products

The enzymatic reaction products of the substrates (including guaiacol, syringol and anisole) were monitored by Ultra Performance Liquid Chromatography(UPLC)-MS. The products were analyzed in a SHIMADZU LC-20AD/AB SCIEX QTRAP 5500 system, using a reverse-phase C18 column (CAPCELL PAK C18 MGII 5.0 μm, 2.0 mm × 150 mm) at 25 °C and a flow rate of 0.5 mL min^−1^. The change of mobile phase (water: eluent A; acetonitrile: eluent B) along time was shown below: 0–2.5 min: 95%A/5%B, 2.6–5 min: 80%A/20%B, 5.1–7.5 min: 50%A/50%B, 7.6–10 min: 10%A/90%B, 10.1–13 min: 95%A/5%B. The mass spectrometer was operated in the negative ion mode.

#### 3.2.6. ^1^ H NMR Spectroscopy

Next, we isolated the *O*-demethylation product of guaiacol, syringol and anisole. The solution after the reaction was extracted with trichloromethane, which was then separated by a TLC plate (20 cm × 20 cm; 1.5 mm thickness) using a solvent system of petroleum ether and ethyl acetate (*v*/*v* =5:1). The chromatogram was visualized under 254 nm, and the corresponding dot on the plate was scratched and dissolved in HPLC grade methanol and centrifuged at 12,000 rpm for 15 min in order to remove silica. The supernatant was collected, filtered through a 0.22 μm filter, and dried under reduced pressure for the ^1^ H NMR experiments. The ^1^H NMR spectra of the products were recorded on a Bruker Advance III 600 MHz spectrometer in CDCl_3_ at 298 K.

## 4. Conclusions

In summary, through the rearrangement of amino acids in the active site, we successfully constructed a highly selective peroxygenase system based on P450BM3 for the *O*-demethylation of various aromatic ethers. Compared with previous P450BM3 mutants that do not possess high activity and selectivity to *O*-demethylation of guaiacol, the combination of F87A/V78A/T268I/A264G and Im-C6-Phe removed this barrier and exhibited TON of 839 to guaiacol with 100% selectivity. From the perspective of selectivity, this TON is approximately eight times higher than that of F87A/T268A (TON: 96), which showed the highest activity in mutants with 100% selectivity of previous study [21]. However, from the perspective of *O*-demethylation activity, the best mutant (F87A/V78A/T268I/A264G) in this study was only 56% higher than F87A (TON: 539, selectivity: 61%), which is the best mutant in previous study [21]. In addition, the mutants devised here also exhibited improved catalytic ability to syringol. Our research has only made phased progress, but the use of H_2_O_2_ as an oxidant to convert lignin monomers has a broad prospect from the economic viewpoint. Further optimization of the P450 peroxygenase system could be realized by protein engineering or by a combination with the in situ formation of H_2_O_2_ to control its instantaneous concentration [26,27,28], which is now underway in our laboratory.

## Figures and Tables

**Table 1:** Oxidation of guaiacol (1) catalyzed by the P450BM3 peroxygenase system ^a^.

Enzyme	DFSM	TON ^b^				Catechol Selectivity %
		1a	1b	1c	1d	
F87A [21]	Im-C6-Phe ^c^	539 ± 2	nd ^d^	nd	344 ± 11	61
F87G [21]	Im-C6-Phe	495 ± 9	nd	nd	236 ± 12	68
F87A/T268L	Im-C6-Phe	10 ± 1	nd	nd	nd	100
F87A/T268P	Im-C6-Phe	321 ± 2	nd	nd	50 ± 2	86
F87A/T268I [21]	Im-C6-Phe	96 ± 1	nd	nd	16 ± 0.1	85
F87A/V78A	Im-C6-Phe	448 ± 18	nd	nd	37 ±3	92
F87G/V78A	Im-C6-Phe	285 ± 12	nd	nd	46 ± 3	86
F87A/V78A/A264G	Im-C6-Phe	333 ± 14	nd	nd	15 ± 1	96
F87G/V78A/A264G	Im-C6-Phe	67 ± 2	nd	nd	2 ± 1	97
F87A/V78A/T268I	Im-C6-Phe	428 ± 4	800 ± 6	18 ± 1	26 ± 1	34
F87A/V78G/T268I	Im-C6-Phe	188 ± 5	521 ± 15	12 ± 1	17 ± 1	25
F87A/V78A/T268P	Im-C6-Phe	174 ± 3	26 ± 1	10 ± 0.6	70 ± 1	62
F87A/V78G/T268P	Im-C6-Phe	353 ± 2	nd	9 ± 1	79 ± 1	80
F87A/T268P/A264G	Im-C6-Phe	269 ± 9	nd	nd	nd	100
F87A/V78A/T268I/A264G	Im-C6-Phe	839 ± 7	nd	nd	nd	100
F87A/V78A/A264G/T268A	Im-C6-Phe	154 ± 5	nd	nd	nd	100

^a^ Reaction conditions: P450BM3 (0.5 μM), substrate (4 mM), H_2_O_2_ (30 mM), DFSM (0.5 mM), in pH 8.0 phosphate buffer. ^b^ TON: turnover number was estimated for 30 min reactions. Average errors are representative of three or more independent measurements. ^c^ Im-C6-Phe: N-(ω-imidazol-1-yl hexanoyl)-L -phenylalanine. ^d^ nd: not detected.

**Table 2:** Oxidation of syringol (2) catalyzed by the P450BM3 peroxygenase system ^a^.

Enzyme	DFSM	TON ^b^		3-Methoxycatechol Selectivity %
		2a	2b	
F87A/T268P	Im-C6-Phe ^c^	238 ± 2	nd ^d^	100
F87A/V78A	Im-C6-Phe	nd	nd	—
F87A/V78G	Im-C6-Phe	30 ± 1	nd	100
F87A/V78A/T268I	Im-C6-Phe	200 ± 1	nd	100
F87A/V78G/T268I	Im-C6-Phe	208 ± 1	nd	100
F87A/T268P/V78A	Im-C6-Phe	236 ± 5	nd	100
F87A/T268P/A264G	Im-C6-Phe	97 ± 12	nd	100
F87A/V78A/T268I/A264G	Im-C6-Phe	67 ± 1	nd	100

^a^ Reaction conditions: P450BM3 (0.5 μM), substrate (4 mM), H_2_O_2_ (30 mM), DFSM (0.5 mM), in pH 8.0 phosphate buffer. ^b^ TON: turnover number estimated for 30-min reactions. Average errors are representative of three or more independent measurements. ^c^ Im-C6-Phe: N-(ω-imidazol-1-yl hexanoyl)-L -phenylalanine. ^d^ nd: not detected.

**Table 3:** Regioselective *O-*demethylation of anisole (3) catalyzed by P450BM3 peroxygenase system ^a^.

Enzyme	DFSM	TON ^b^			Phenol Selectivity %
		3a	3b	3c	
F87A [21]	Im-C6-Phe ^c^	145 ± 3	12 ± 1	393 ± 9	26
F87A/T268I [21]	Im-C6-Phe	262 ± 2	nd ^d^	nd	100
F87A/T268P	Im-C6-Phe	128 ± 3	28 ± 2	333 ± 5	26
F87A/V78A/T268I	Im-C6-Phe	265 ± 6	nd	nd	100
F87A/V78G/T268I	Im-C6-Phe	108 ± 2	nd	nd	100
F87A/V78A/T268I/A264G	Im-C6-Phe	67 ± 3	nd	nd	100
F87A/V78A/T268I/A264S	Im-C6-Phe	132 ± 6	nd	nd	100

^a^ Reaction conditions: Reaction conditions: P450BM3 (0.5 μM), substrate (4 mM), H_2_O_2_ (30 mM), DFSM (0.5 mM), in pH 8.0 phosphate buffer. ^b^ TON: Turnover number were estimated for 30-min reactions. Average errors are representative of three or more independent measurements. ^c^ Im-C6-Phe: N-(ω-imidazol-1-yl hexanoyl)-L-phenylalanine. ^d^ nd: not detected.

## Data Availability

All data included in this study are available upon request by contact with the corresponding authors.

## Supplementary Materials

The following supporting information can be downloaded at: https://www.mdpi.com/article/10.3390/molecules27103120/s1. Figure S1. HPLC analyses for the oxidation of guaiacol catalyzed by P450BM3 and its mutants in the presence of DFSM; Figure S2. HPLC analyses for the oxidation of 2,6-dimethoxyphenol catalyzed by P450BM3 and its mutants in the presence of DFSM; Figure S3. HPLC analyses for the oxidation of anisole by P450BM3 and its mutants in the presence (left column) of DFSM; Figure S4. SDS-PAGE of P450BM3 and its mutants; Figure S5. UV-visible spectral changes of the wild type P450BM3 and its mutants (red line) upon addition of Na2S2O4 (black line) for the formation of a ferrous CO complex through the reduction of ferric heme; Figure S6. 1 H-NMR (600 MHz) spectrum of the O-demethylation product of guaiacol in CDCl3 at room temperature. Figure S7. 1 H-NMR (600 MHz) spectrum of the O-demethylation product of syringol in CDCl3 at room temperature; Figure S8. 1 H-NMR (600 MHz) spectrum of the O-demethylation product of anisole in CDCl3 at room temperature; Table S1. Oxidation of guaiacol catalysed by P450BM3 peroxygenase system; Table S2. Oxidation of 2,6-dimethoxyphenol catalysed by P450BM3 peroxygenase system; Table S3. Oxidation of anisole catalysed by P450BM3 peroxygenase system; Table S4. Primers used in mutagenesis.
