# Supplementary material for: Protein Engineering of an Artificial P450BM3 Peroxygenase System Enables Highly Selective O-Demethylation of Lignin Monomers
Source: Molecules. 2022 May 13;27(10):3120. doi: 10.3390/molecules27103120 (PMC9143554; doi:10.3390/molecules27103120)
Supplement: Supplementary file 1 [file molecules-27-03120-s001.zip › molecules-1696765-supplementary.pdf]

Supplementary Material

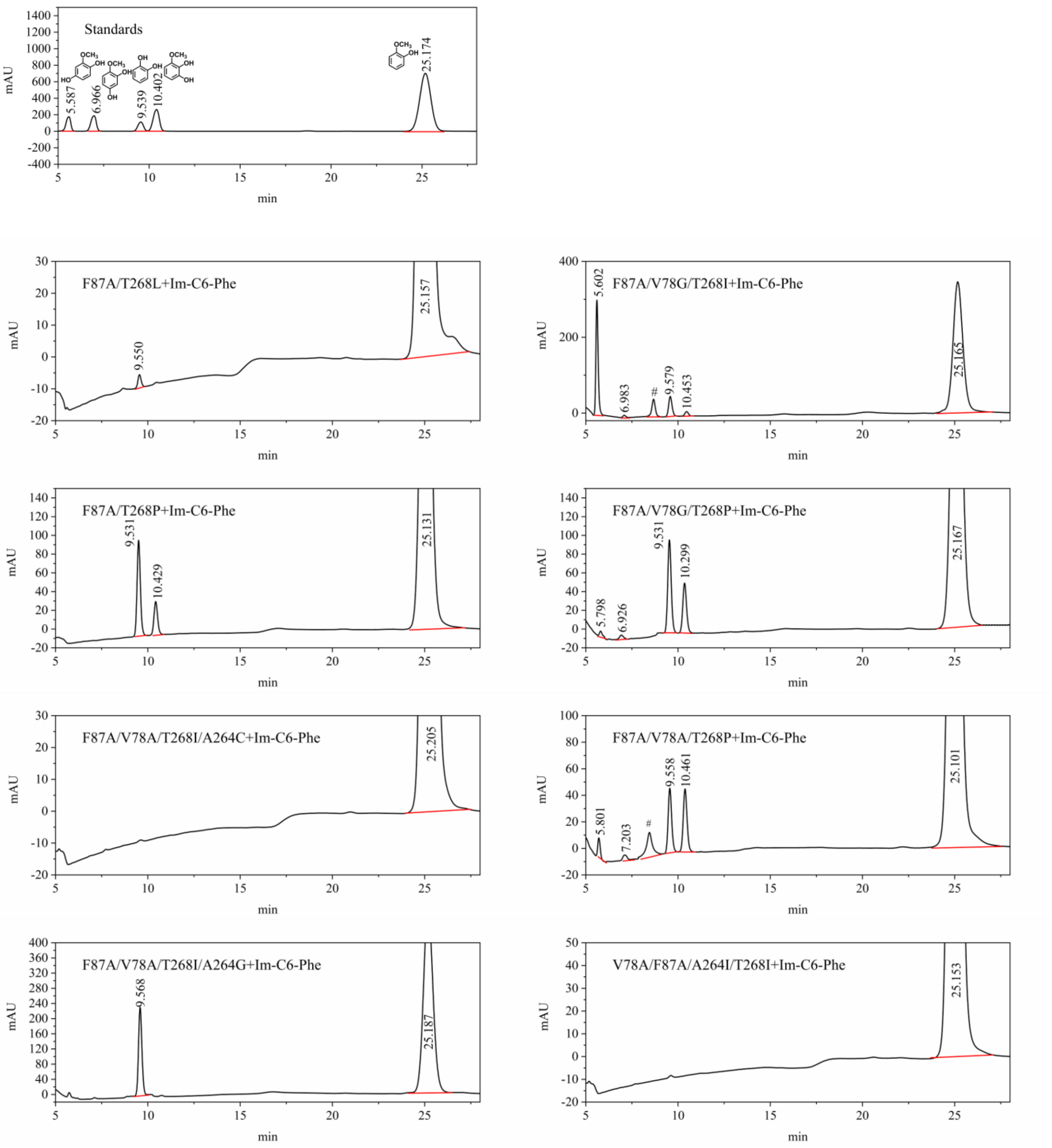

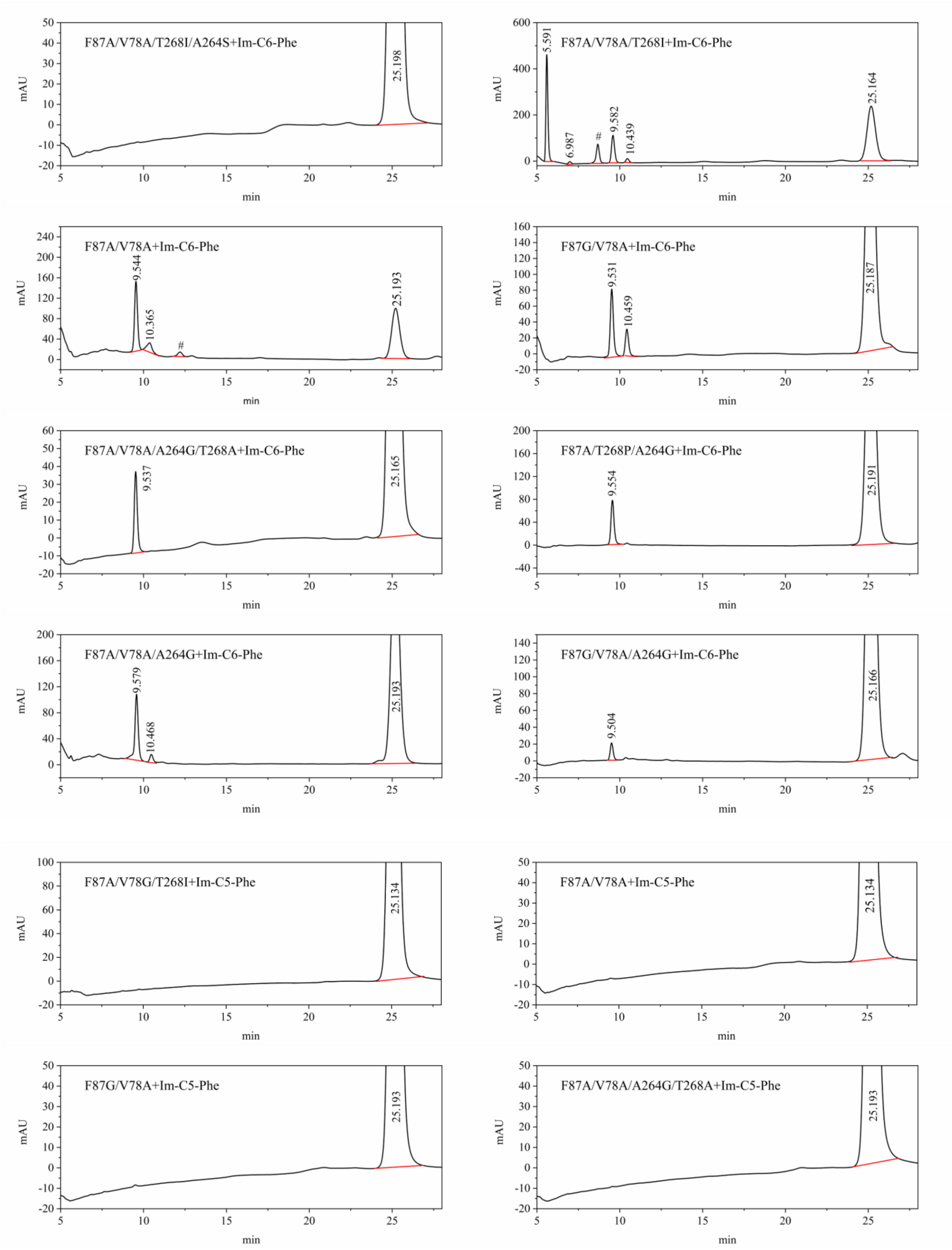
F87A/V78A/T268I/A264S+Im-C6-Phe
25.198
F87A/V78A/T268I+Im-C6-Phe
5.591
6.987
#
9.582
10.439
25.164
F87A/V78A+Im-C6-Phe
9.544
10.365
#
25.193
F87G/V78A+Im-C6-Phe
9.531
10.459
25.187
F87A/V78A/A264G/T268A+Im-C6-Phe
9.537
25.165
F87A/T268P/A264G+Im-C6-Phe
9.554
25.191
F87A/V78A/A264G+Im-C6-Phe
9.579
10.468
25.193
F87G/V78A/A264G+Im-C6-Phe
9.504
25.166
F87A/V78G/T268I+Im-C5-Phe
25.134
F87A/V78A+Im-C5-Phe
25.134
F87G/V78A+Im-C5-Phe
25.193
F87A/V78A/A264G/T268A+Im-C5-Phe
25.193
mAU
min

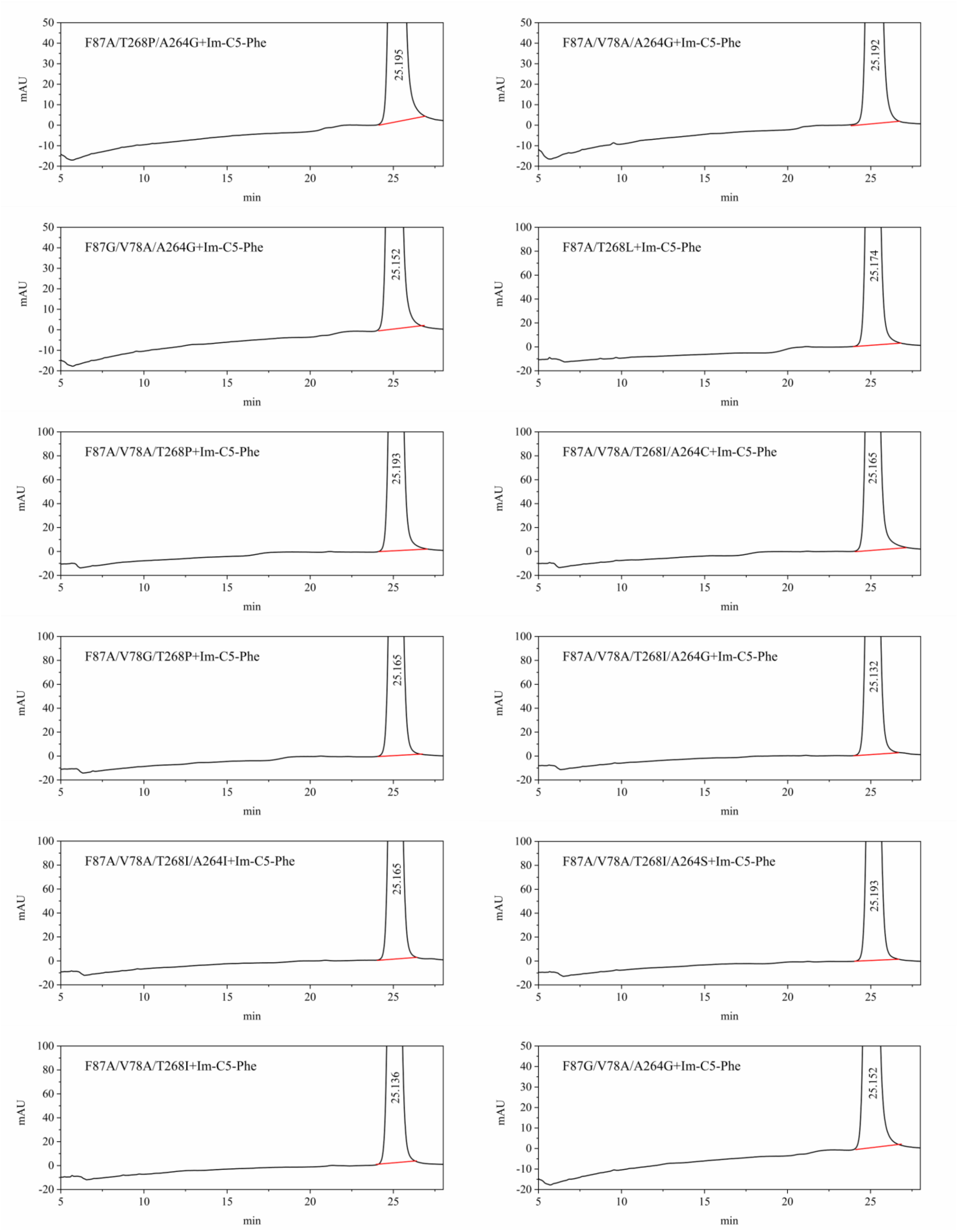

Figure S1. HPLC analyses for the oxidation of guaiacol catalyzed by P450BM3 and its mutants in the presence of DFSM. *The top HPLC spectrum is for the mixed standard of 2-methoxyhydroqui-

none (5.587 min), 4methoxybenzene-1,3-diol (6.966 min), catechol (9.539 min), 3-methoxycatechol (10.402 min) and guaiacol (25.174 min). The peaks labelled as “#” are from uncertain products.

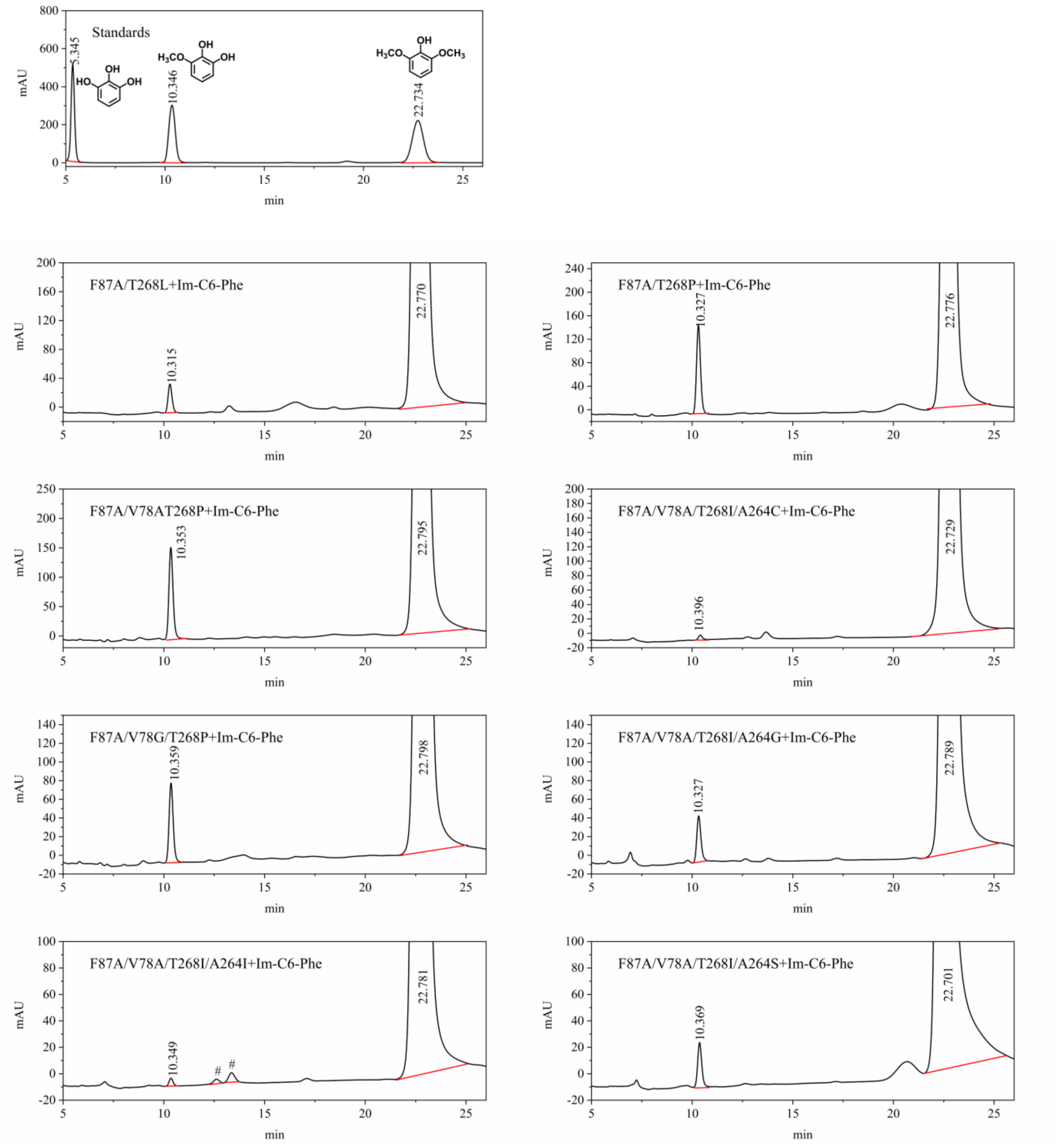

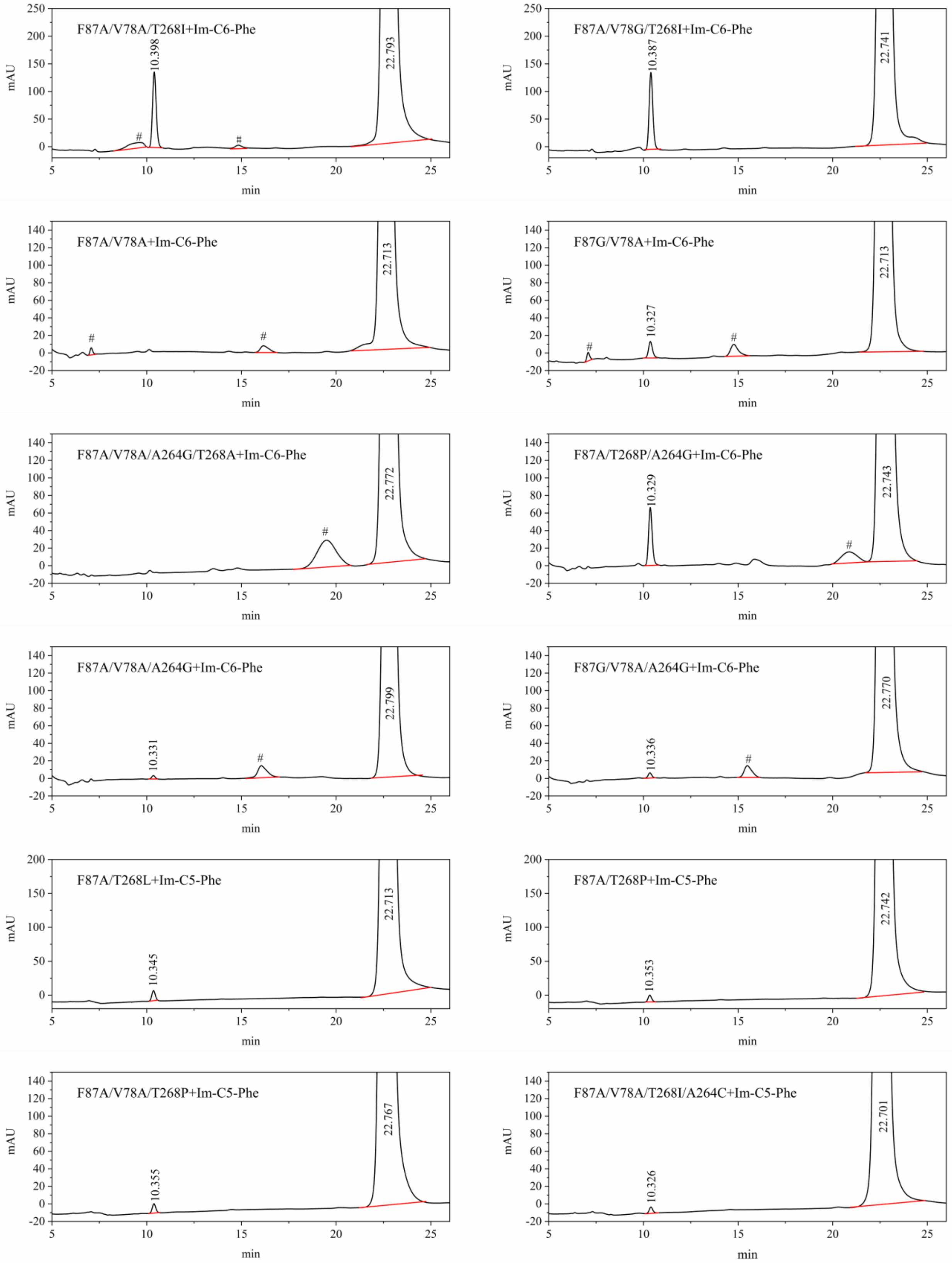
F87A/V78A/T268I+Im-C6-Phe
10.398
22.793
F87A/V78G/T268I+Im-C6-Phe
10.387
22.741
F87A/V78A+Im-C6-Phe
22.713
F87G/V78A+Im-C6-Phe
10.327
22.713
F87A/V78A/A264G/T268A+Im-C6-Phe
22.772
F87A/T268P/A264G+Im-C6-Phe
10.329
22.743
F87A/V78A/A264G+Im-C6-Phe
10.331
22.799
F87G/V78A/A264G+Im-C6-Phe
10.336
22.770
F87A/T268L+Im-C5-Phe
10.345
22.713
F87A/T268P+Im-C5-Phe
10.353
22.742
F87A/V78A/T268P+Im-C5-Phe
10.355
22.767
F87A/V78A/T268I/A264C+Im-C5-Phe
10.326
22.701
mAU
min

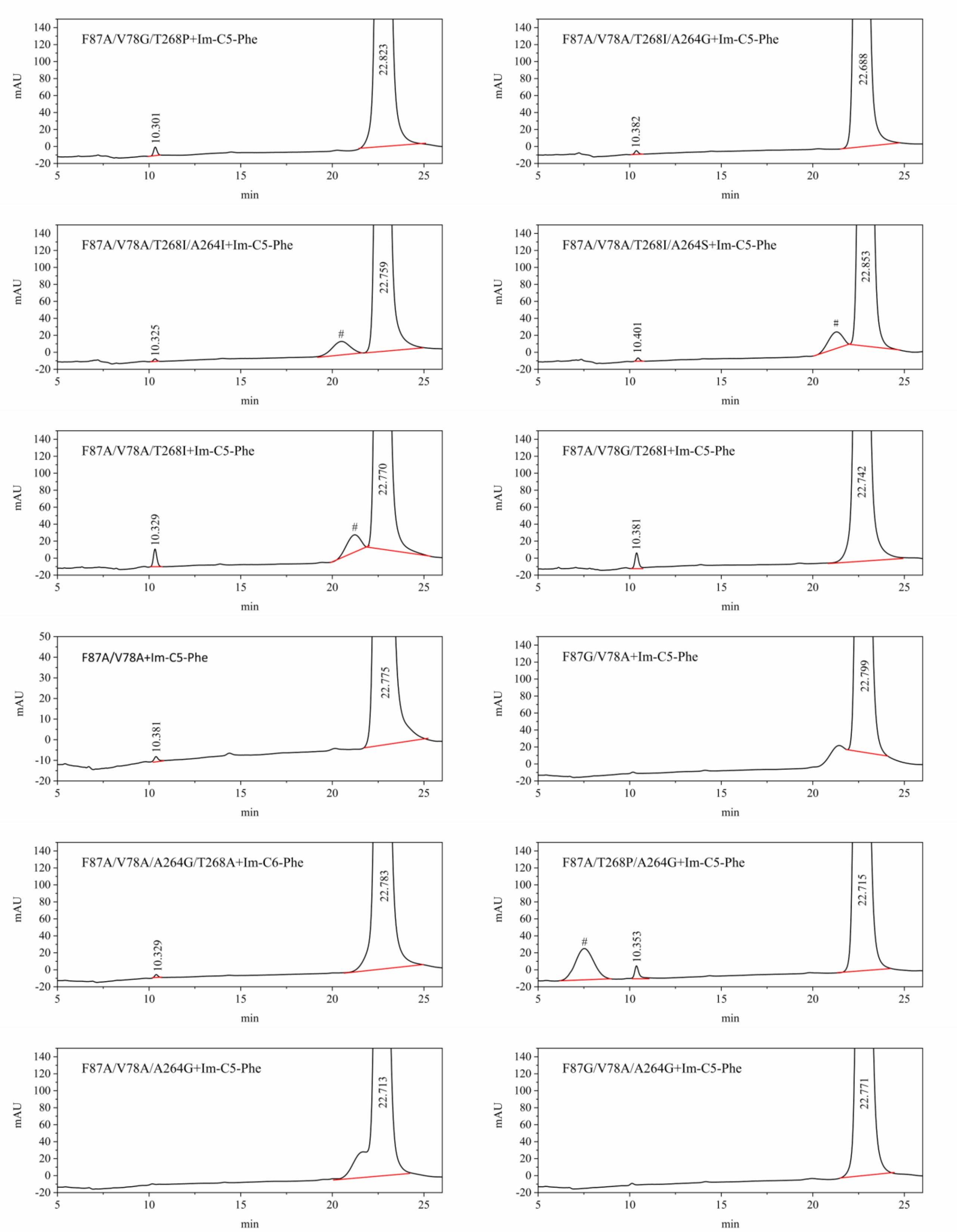
F87A/V78G/T268P+Im-C5-Phe
10.301
22.823
F87A/V78A/T268I/A264G+Im-C5-Phe
10.382
22.688
F87A/V78A/T268I/A264I+Im-C5-Phe
10.325
#
22.759
F87A/V78A/T268I/A264S+Im-C5-Phe
10.401
#
22.853
F87A/V78A/T268I+Im-C5-Phe
10.329
#
22.770
F87A/V78G/T268I+Im-C5-Phe
10.381
22.742
F87A/V78A+Im-C5-Phe
10.381
22.775
F87G/V78A+Im-C5-Phe
22.799
F87A/V78A/A264G/T268A+Im-C6-Phe
10.329
22.783
F87A/T268P/A264G+Im-C5-Phe
#
10.353
22.715
F87A/V78A/A264G+Im-C5-Phe
22.713
F87G/V78A/A264G+Im-C5-Phe
22.771
mAU
min

Figure S2. HPLC analyses for the oxidation of 2,6-Dimethoxyphenol catalyzed by P450BM3 and its mutants in the presence of DFSM. *The top HPLC spectrum is for the mixed standard of pyrogallol (5.345 min), 3- methoxycatechol (10.346 min) and 2,6-dimethoxyphenol (22.734 min). The peaks labelled as “#” are from uncertain products.

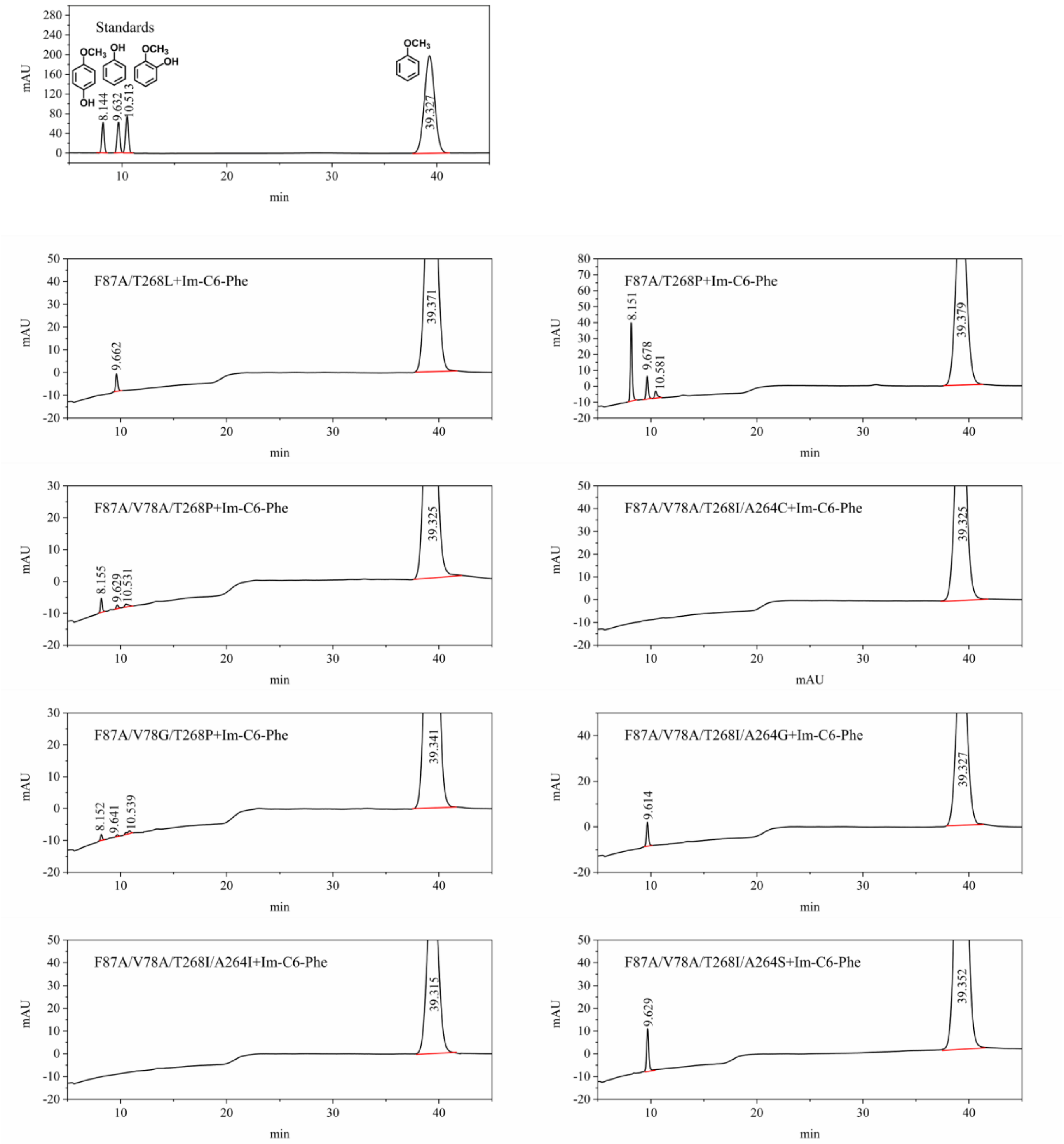

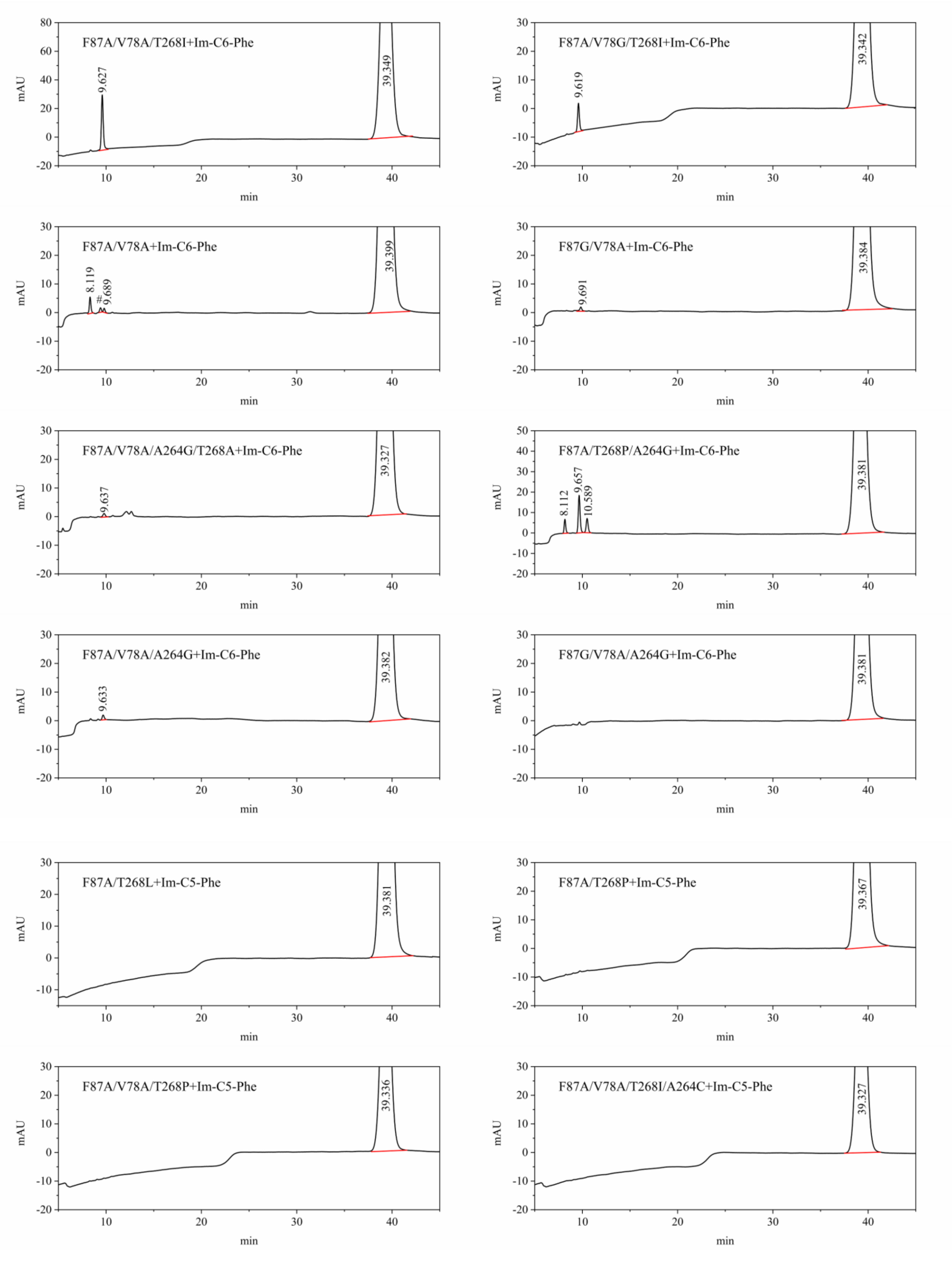
F87A/V78A/T268I+Im-C6-Phe
9.627
39.349
F87A/V78G/T268I+Im-C6-Phe
9.619
39.342
F87A/V78A+Im-C6-Phe
8.119
#
9.689
39.399
F87G/V78A+Im-C6-Phe
9.691
39.384
F87A/V78A/A264G/T268A+Im-C6-Phe
9.637
39.327
F87A/T268P/A264G+Im-C6-Phe
8.112
9.657
10.589
39.381
F87A/V78A/A264G+Im-C6-Phe
9.633
39.382
F87G/V78A/A264G+Im-C6-Phe
39.381
F87A/T268L+Im-C5-Phe
39.381
F87A/T268P+Im-C5-Phe
39.367
F87A/V78A/T268P+Im-C5-Phe
39.336
F87A/V78A/T268I/A264C+Im-C5-Phe
39.327
mAU
min

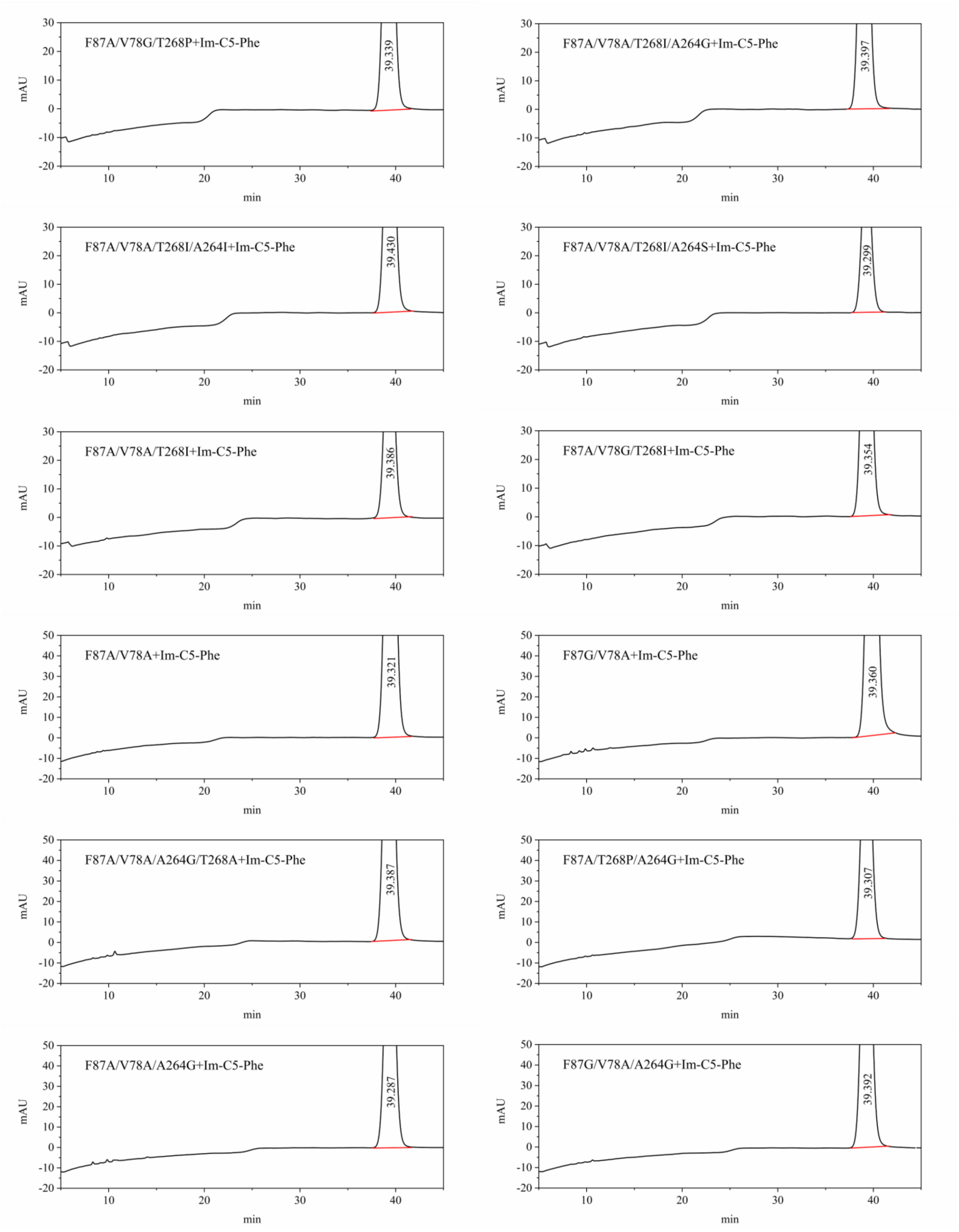

Figure S3. HPLC analyses for the oxidation of anisole (1) by P450BM3 and its mutants in the absence (right column) /presence (left column) of DFSM. The top HPLC spectrum is for the mixed

standard of 4-methoxyphenol (8.144 min), Phenol (9.632 min), guaiacol (10.513 min) and anisole (39.327 min). The peaks labelled as "#" are from uncertain products.

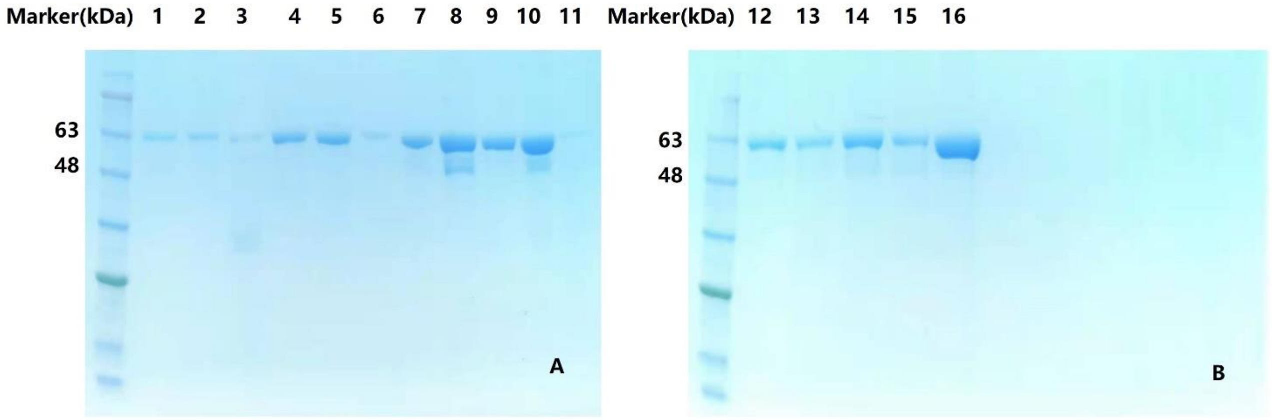

Figure S4. SDS-PAGE of P450BM3 and its mutants. (A) Lane 1-11: F87A/T268L, F87A/T268P, F87A/V78A, F87G/V78A, F87A/V78A/A264G, F87G/V78A/A264G, F87A/V78A/T268I, F87A/V78G/T268I, F87A/V78A/T268P, F87A/V78G/T268P, F87A/T268P/A264G; (B) Lane 12-16: F87A/V78A/T268I/A264G, F87A/V78A/T268I/A264C, F87A/V78A/T268I/A264S, F87A/V78A/T268I/A264I, F87A/V78A/A264G/T268A

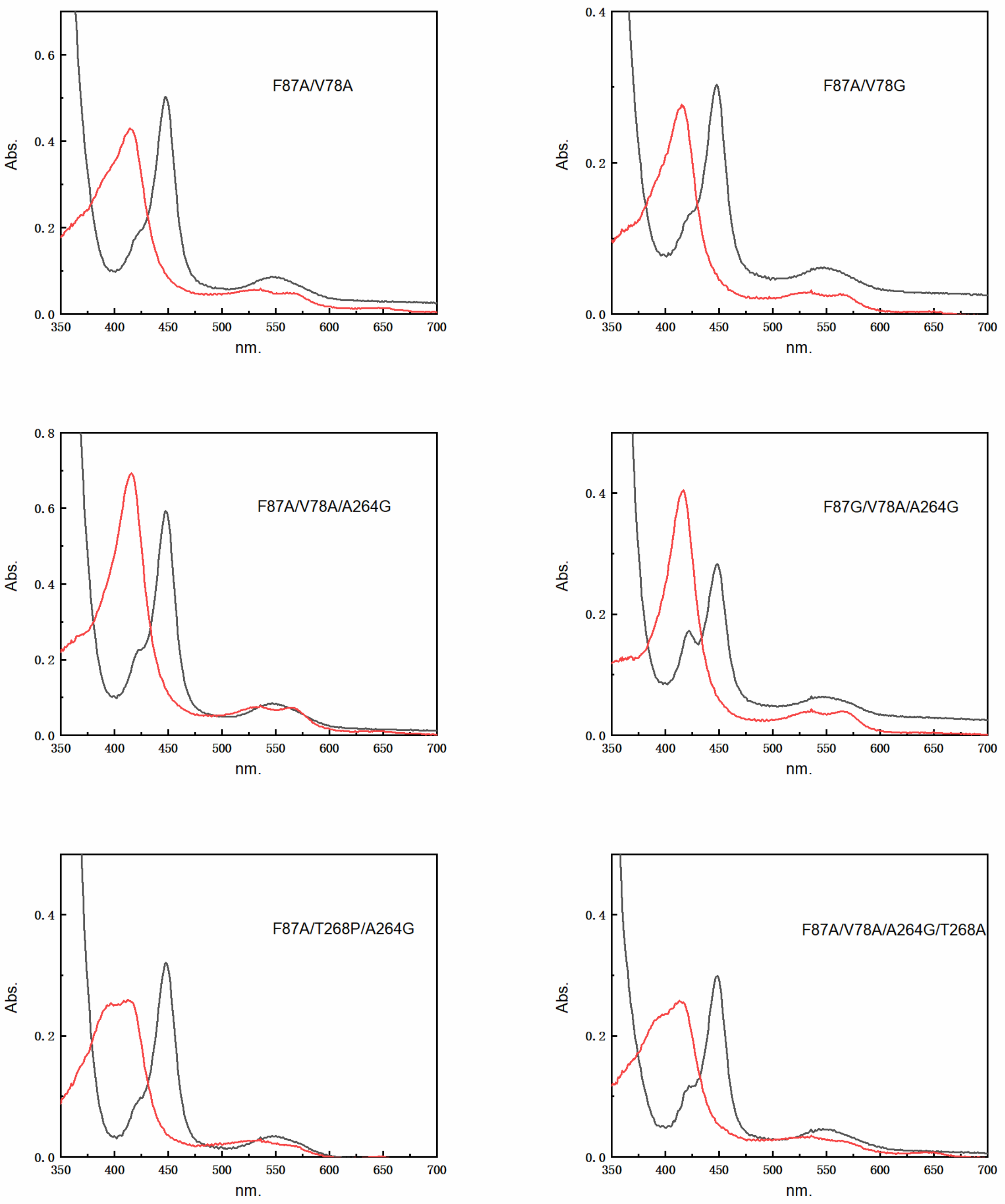
F87A/V78A
F87A/V78G
F87A/V78A/A264G
F87G/V78A/A264G
F87A/T268P/A264G
F87A/V78A/A264G/T268A
Abs.
nm.
350
400
450
500
550
600
650
700
0.0
0.2
0.4
0.6
0.8

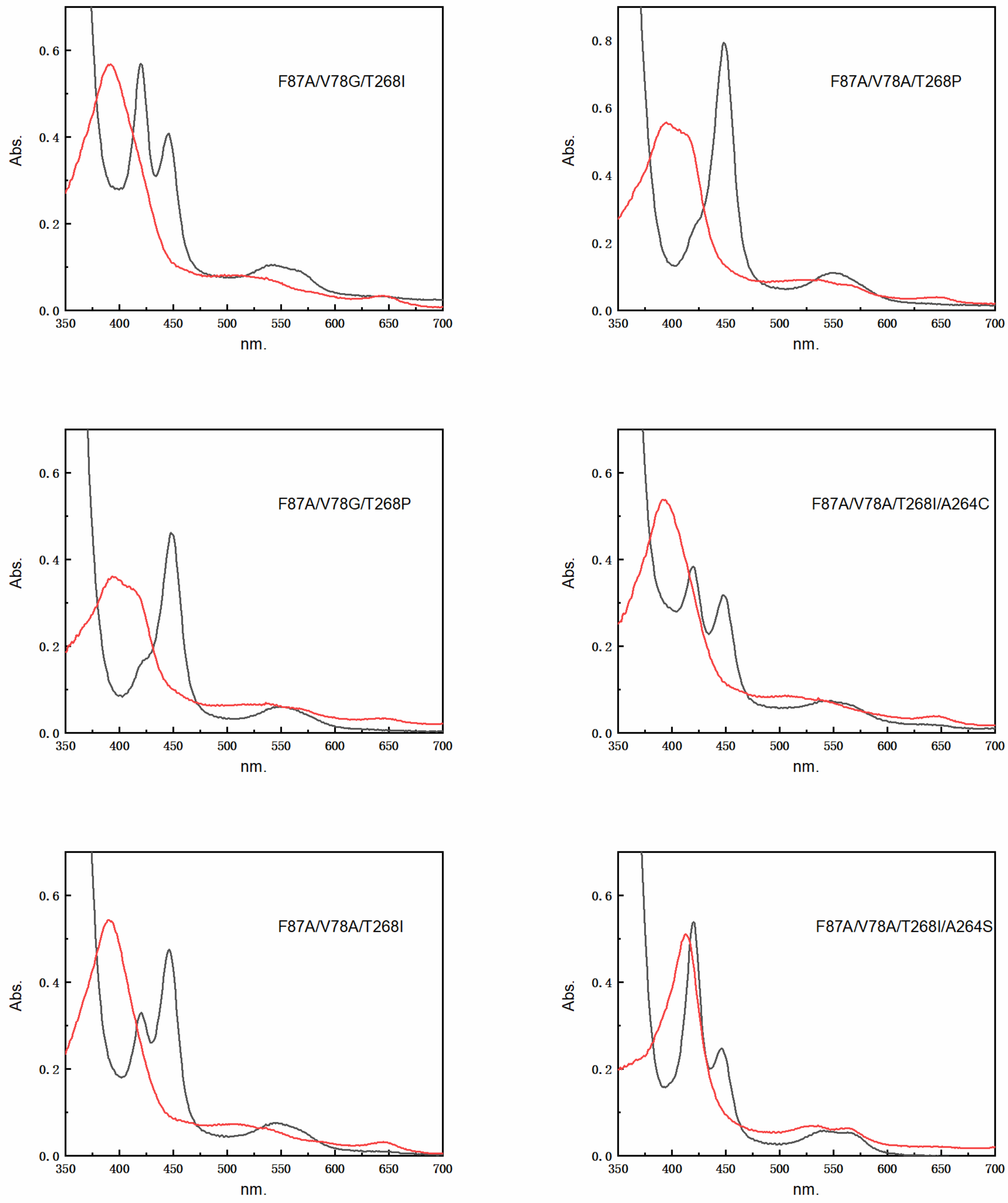
F87A/V78G/T268I
F87A/V78A/T268P
F87A/V78G/T268P
F87A/V78A/T268I/A264C
F87A/V78A/T268I
F87A/V78A/T268I/A264S
Abs.
nm.

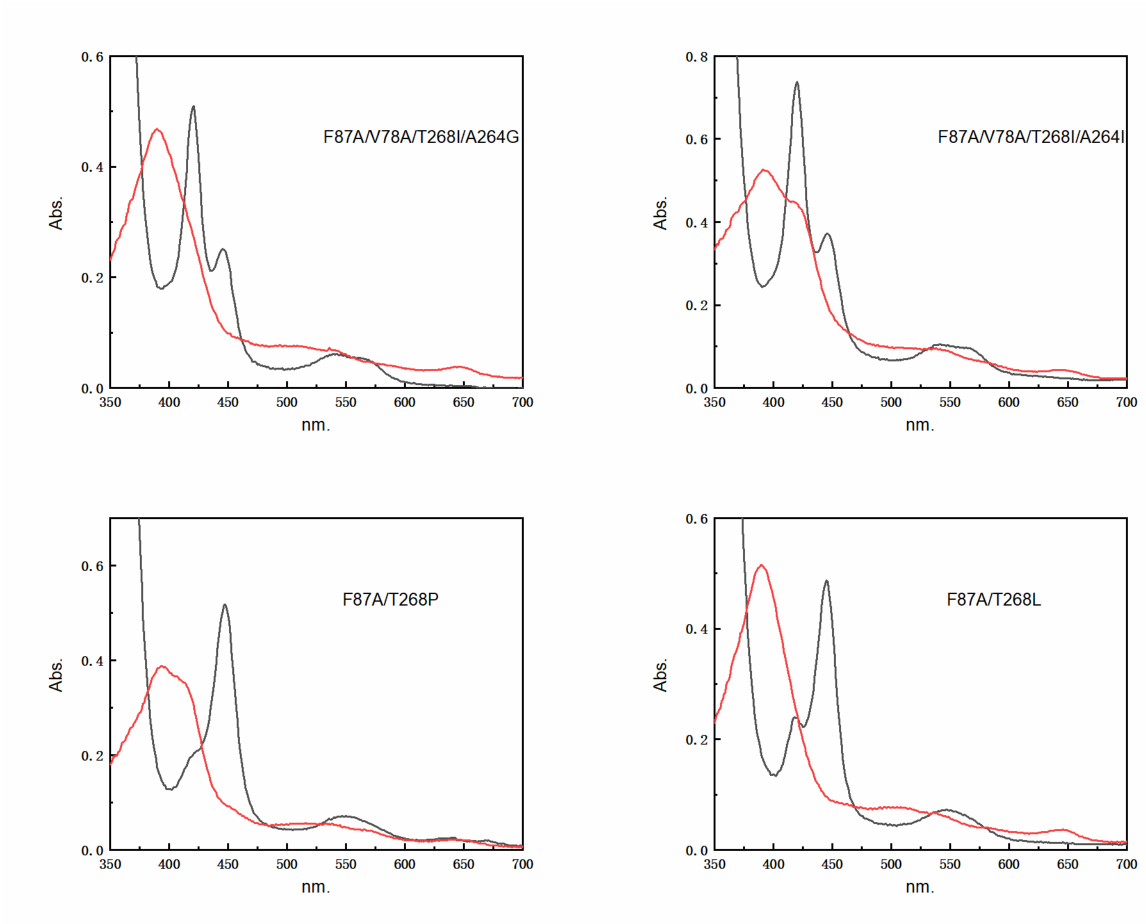

Figure S5. UV-visible spectral changes of the wild type P450BM3 and its mutants (red line) upon addition of $Na_2S_2O_4$ (black line) for the formation of a ferrous CO complex through the reduction of ferric heme.

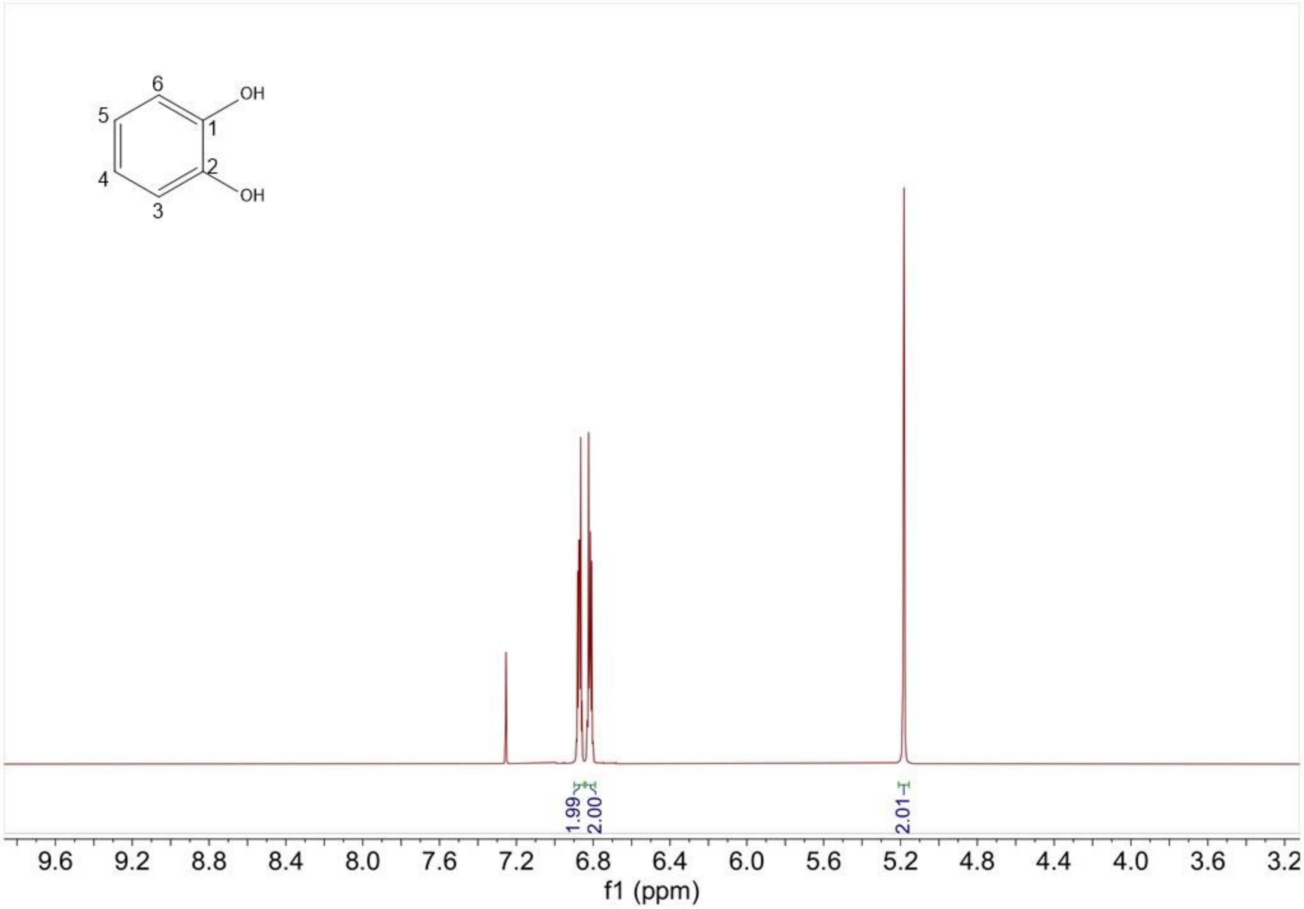

Figure S6 ^{1}H-NMR (600 MHz) spectrum of the *O*-demethylation product of guaiacol in $CDCl_3$ at room temperature. δ (ppm) = 6.89 - 6.86 (m, 2H, H_4, H_5), 6.83 - 6.80 (m, 2H, H_3, H_6), 5.18 (s, 2H, H_{OH1}, H_{OH2}).

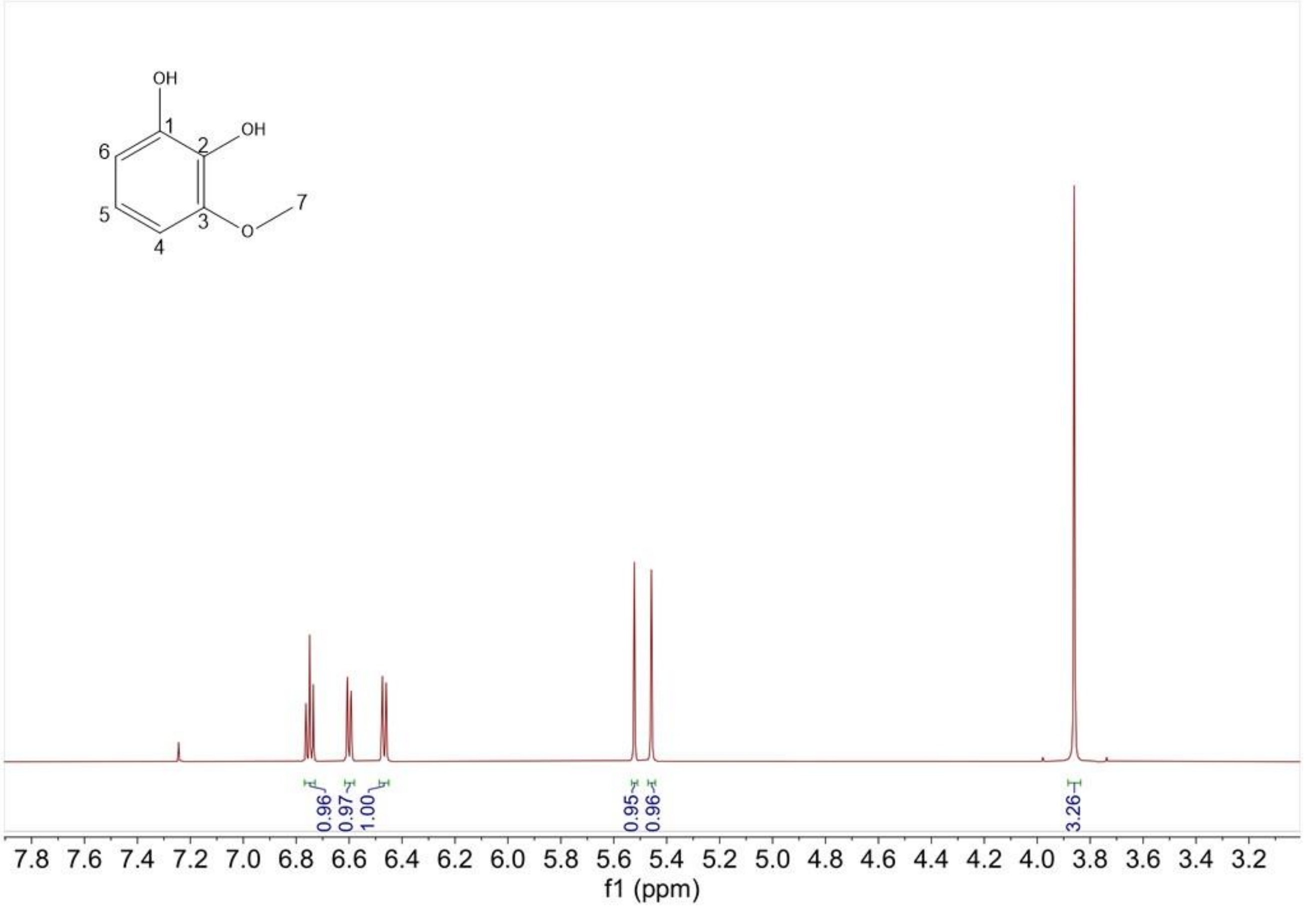

Figure S7 ^{1}H-NMR (600 MHz) spectrum of the *O*-demethylation product of syringol in $CDCl_3$ at room temperature. δ（ppm) = 6.77 - 6.73 (m, 1H, H_5), 6.60 (dd, *J* = 8.28, 1.02 Hz, 1H, H_6), 6.49 - 6.45 (dd, *J* = 8.28, 1.02 Hz, 1H, H_4), 5.52 (s, 1H, H_{OH1}), 5.46 (s, 1H, H_{OH2}), 3.86 (s, 3H, H_7).

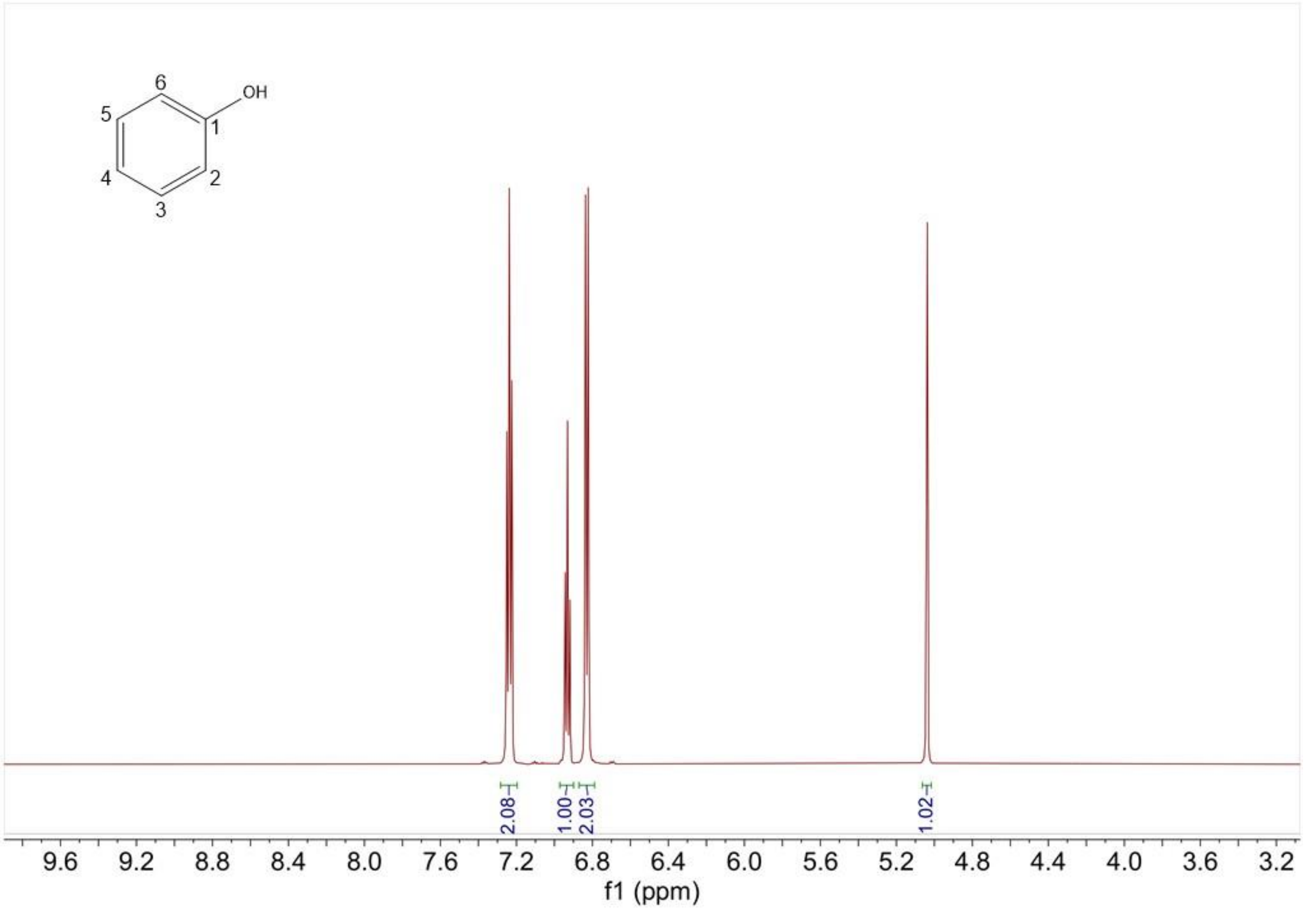

Figure S8 ^{1}H-NMR (600 MHz) spectrum of the *O*-demethylation product of anisole in $CDCl_3$ at room temperature. δ (ppm) = 7.26 - 7.21 (m, 2H, H3, H5), 6.95 - 6.91 (m, 1H, H4), 6.85 - 6.81 (m, 2H, H2, H6), 5.04 (s, 1H, H_{OH}).

Table S1. Oxidation of guaiacol catalysed by P450BM3 peroxygenase system[a]

Enzyme	DFSM	TON[b]				Catechol Selectivity %
		1a	1b	1c	1d	
F87A/T268L	Im-C6-Phe[c]	10 ± 1	nd[d]	nd	nd	100
F87A/T268P	Im-C6-Phe	321 ± 2	nd	nd	50 ± 2	86
F87A/V78A	Im-C6-Phe	448 ± 18	nd	nd	37 ±3	92
F87G/V78A	Im-C6-Phe	285 ± 12	nd	nd	46 ± 3	86
F87A/V78A/A264G	Im-C6-Phe	333 ± 14	nd	nd	15 ± 1	96
F87G/V78A/A264G	Im-C6-Phe	67 ± 2	nd	nd	2 ± 1	97
F87A/V78A/T268I	Im-C6-Phe	428 ± 4	800 ± 6	18 ± 1	26 ± 1	34
F87A/V78G/T268I	Im-C6-Phe	188 ± 5	521 ± 15	12 ± 1	17 ± 1	25
F87A/V78A/T268P	Im-C6-Phe	174 ± 3	26 ± 1	10 ± 0.6	70 ± 1	62
F87A/V78G/T268P	Im-C6-Phe	353 ± 2	nd	9 ± 1	79 ± 1	80
F87A/T268P/A264G	Im-C6-Phe	269 ± 9	nd	nd	nd	100
F87A/V78A/T268I/ A264G	Im-C6-Phe	839 ± 7	nd	nd	nd	100
F87A/V78A/T268I/ A264C	Im-C6-Phe	nd	nd	nd	nd	0
F87A/V78A/T268I/ A264S	Im-C6-Phe	nd	nd	nd	nd	0
F87A/V78A/T268I/ A264I	Im-C6-Phe	nd	nd	nd	nd	0
F87A/V78A/A264G/ T268A	Im-C6-Phe	154 ± 5	nd	nd	nd	100
P450BM3[e]	Im-C5-Phe[f]	nd	nd	nd	nd	—

[a] Reaction conditions: P450BM3 (0.5 μM), substrate (4 mM), H2O2 (30 mM), DFSM (0.5 mM), in pH 8.0 phosphate buffer. [b] TON: turnover number was estimated for 30 minute reactions. Average errors are representative of three or more independent measurements. [c] Im-C6-Phe: N-(ω-imidazol-1-yl hexanoyl)-L -phenylalanine. [d] nd: not detected.[e] All P450-BM3 mutants in this table. [f] Im-C5-Phe: N-(ω-imidazol-1-yl pentanoyl)-L-phenylalanine.

Table S2. Oxidation of 2,6-dimethoxyphenol catalysed by P450BM3 peroxyg-enase system[a]

Enzyme	DFSM	TON[b]
		1a
F87A/T268L	Im-C6-Phe[c]	55 ± 2
F87A/T268L	Im-C5-Phe[d]	17 ± 2
F87A/T268P	Im-C6-Phe	238 ± 2
F87A/T268P	Im-C5-Phe	7 ± 1
F87A/V78A	Im-C6-Phe	nd[e]
F87A/V78A	Im-C5-Phe	3 ± 1
F87G/V78A	Im-C6-Phe	30 ± 1

F87G/V78A	Im-C5-Phe	3 ± 1
F87A/V78A/A264G	Im-C6-Phe	6 ± 1
F87A/V78A/A264G	Im-C5-Phe	nd
F87G/V78A/A264G	Im-C6-Phe	9 ± 1
F87G/V78A/A264G	Im-C5-Phe	3 ± 1
F87A/T268I/V78A	Im-C6-Phe	200 ± 1
F87A/T268I/V78A	Im-C5-Phe	35 ± 1
F87A/T268I/V78G	Im-C6-Phe	208 ± 1
F87A/T268I/V78G	Im-C5-Phe	23 ± 2
F87A/T268P/V78A	Im-C6-Phe	236 ± 5
F87A/T268P/V78A	Im-C5-Phe	7 ± 1
F87A/T268P/V78G	Im-C6-Phe	126 ± 4
F87A/T268P/V78G	Im-C5-Phe	9 ± 1
F87A/T268P/A264G	Im-C6-Phe	97 ± 12
F87A/T268P/A264G	Im-C5-Phe	26 ± 2
F87A/T268I/V78A/A264G	Im-C6-Phe	73 ± 3
F87A/T268I/V78A/A264G	Im-C5-Phe	6 ± 1
F87A/T268I/V78A/A264C	Im-C6-Phe	2 ± 1
F87A/T268I/V78A/A264C	Im-C5-Phe	4 ± 1
F87A/T268I/V78A/A264S	Im-C6-Phe	45 ± 1
F87A/T268I/V78A/A264S	Im-C5-Phe	10 ± 2
F87A/T268I/V78A/A264I	Im-C6-Phe	2 ± 1
F87A/T268I/V78A/A264I	Im-C5-Phe	5 ± 1
F87A/V78A/A264G/T268A	Im-C6-Phe	nd
F87A/V78A/A264G/T268A	Im-C5-Phe	5 ± 1

[a] Reaction conditions: P450BM3 (0.5 μM), substrate (4 mM), H2O2 (30 mM), DFSM (0.5 mM), in pH 8.0 phosphate buffer. [b] TON: turnover number was estimated for 30 minute reactions. Average errors are representative of three or more independent measurements. [c] Im-C6-Phe: N-(ω-imidazol-1-yl hexanoyl)-L -phenylalanine. [d] Im-C5-Phe: N-(ω-imidazol-1-yl pentanoyl)-L-phenylalanine. [e] nd: not detected.

Table S3. Oxidation of anisole catalysed by P450BM3 peroxygenase system[a]

Enzyme	DFSM	TON[b]			Phenol selectivity %
		3a	3b	3c	
F87A[16]	Im-C6-Phe[c]	145 ± 3	12 ± 1	393 ± 9	26
F87A/T268I[16]	Im-C6-Phe	262 ± 2	nd[d]	nd	100
F87A/T268L	Im-C6-Phe	42 ± 2	nd	nd	100
F87A/T268P	Im-C6-Phe	128 ± 3	28 ± 2	333 ± 5	26
F87A/V78A	Im-C6-Phe	5 ± 2	nd	24 ± 2	17
F87G/V78A	Im-C6-Phe	6 ± 1	nd	nd	100
F87A/V78A/A264G	Im-C6-Phe	8 ± 1	nd	nd	100
F87G/V78A/A264G	Im-C6-Phe	nd	nd	nd	-
F87A/V78A/T268I	Im-C6-Phe	265 ± 6	nd	nd	100
F87A/V78G/T268I	Im-C6-Phe	108 ± 2	nd	nd	100
F87A/V78A/T268P	Im-C6-Phe	11 ± 2	4 ± 1	36 ± 3	22
F87A/V78G/T268P	Im-C6-Phe	6 ± 1	7 ± 1	16 ± 2	21
F87A/T268P/A264G	Im-C6-Phe	80 ± 3	24 ± 1	27 ± 1	61
F87A/V78A/T268I/A264G	Im-C6-Phe	67 ± 3	nd	nd	100
F87A/V78A/T268I/A264C	Im-C6-Phe	nd	nd	nd	-
F87A/V78A/T268I/A264S	Im-C6-Phe	132 ± 6	nd	nd	100
F87A/V78A/T268I/A264I	Im-C6-Phe	nd	nd	nd	-
F87A/V78A/A264G/T268A	Im-C6-Phe	7 ± 1	nd	nd	100
P450BM3[e]	Im-C5-Phe[f]	nd	nd	nd	-

[a] Reaction conditions: Reaction conditions: P450BM3 (0.5 μM), substrate (4 mM), H2O2 (30 mM), DFSM (0.5 mM), in pH 8.0 phosphate buffer. [b] TON: Turnover number were estimated for 30-minutes reactions. Average errors are representative of three or more independent measurements. [c] Im-C6-Phe: N-(ω-imidazol-1-yl hexanoyl)-L-phenylalanine. [d] nd: not detected. [e] All prepared P450BM3 mutants in the paper. [f] Im-C5-Phe: N-(ω-imidazol-1-yl pentanoyl)-L-phenylalanine

Table S4. Primers used in mutagenesis

Parent template	primer	sequence（5’- 3’）
F87A	V78A-F	CTTAAATTTGCACGTGATTTTGCAG
	V78-R	CGCTTGACTTAAGTTTTTATC
	T268L-F	GGGACACGAATTAACAAGTGGTC
	T268P-F	GGGACACGAACCTACAAGTGGTC
	T268-R	GCAATTAAGAATGTAATAATTTGATAG
F87G	V78A-F	CTTAAATTTGCACGTGATTTTGCAG
	V78-R	CGCTTGACTTAAGTTTTTATC
F87A/V78A	A264G-F	ATTCTTAATTGGAGGACACGAAACAAC
	T268I-F	GGGACACGAAATCACAAGTGGTC
	T268P-F	GGGACACGAACCTACAAGTGGTC
	A264-R	GTAATAATTTGATAGCGAATGTTC
	T268-R	GCAATTAAGAATGTAATAATTTGATAG
F87A/V78G	T268I-F	GGGACACGAAATCACAAGTGGTC
	T268P-F	GGGACACGAACCTACAAGTGGTC
	T268-R	GCAATTAAGAATGTAATAATTTGATAG

F87G/V78A	A264G-F	ATTCTTAATTGGAGGACACGAAACAAC
	A264-R	GTAATAATTTGATAGCGAATGTTC
F87A/T268P	A264G-F	ATTCTTAATTGGAGGACACGAACCTAC
	A264-R	GTAATAATTTGATAGCGAATGTTC
F87A/V78A/T268I	A264G-F	ATTCTTAATTGGAGGACACGAAATCAC
	A264C-F	ATTCTTAATTTGCGGACACGAAATCACAAGTG
	A264S-F	ATTCTTAATTTCAGGACACGAAATCACAAG
	A264I-F	ATTCTTAATTATTGGACACGAAATCACAAG
	A264-R	GTAATAATTTGATAGCGAATGTTC
F87A/V78A/A264G/T28I	I268A-F	AGGACACGAAGCGACAAGTGGTC
	I268-R	CCAATTAAGAATGTAATAATTTGATAG
